# Evidence for widespread existence of functional novel and non-canonical human transcripts

**DOI:** 10.1186/s12915-023-01753-5

**Published:** 2023-11-24

**Authors:** Dongyang Xu, Lu Tang, Junjun Zhou, Fang Wang, Huifen Cao, Yu Huang, Philipp Kapranov

**Affiliations:** 1 Institute of Genomics, School of Medicine, Huaqiao University, 668 Jimei Road, Xiamen, 361021 China; 2 State Key Laboratory of Cellular Stress Biology, School of Life Sciences, Xiamen University, Xiamen, 361102 China

**Keywords:** Insertional mutagenesis, Functional genomics, RNA dark matter, Non-canonical transcript, Novel exon, Alternative splicing, Bi-cistronic mRNA, Transcript targeting technique, Exon prediction, Rapid amplification of cDNA ends

## Abstract

**Background:**

Fraction of functional sequence in the human genome remains a key unresolved question in Biology and the subject of vigorous debate. While a plethora of studies have connected a significant fraction of human DNA to various biochemical processes, the classical definition of function requires evidence of effects on cellular or organismal fitness that such studies do not provide. Although multiple high-throughput reverse genetics screens have been developed to address this issue, they are limited to annotated genomic elements and suffer from non-specific effects, arguing for a strong need to develop additional functional genomics approaches.

**Results:**

In this work, we established a high-throughput lentivirus-based insertional mutagenesis strategy as a forward genetics screen tool in aneuploid cells. Application of this approach to human cell lines in multiple phenotypic screens suggested the presence of many yet uncharacterized functional elements in the human genome, represented at least in part by novel exons of known and novel genes. The novel transcripts containing these exons can be massively, up to thousands-fold, induced by specific stresses, and at least some can represent bi-cistronic protein-coding mRNAs.

**Conclusions:**

Altogether, these results argue that many unannotated and non-canonical human transcripts, including those that appear as aberrant splice products, have biological relevance under specific biological conditions.

**Supplementary Information:**

The online version contains supplementary material available at 10.1186/s12915-023-01753-5.

## Background

Since the original publication of the human genome sequence [1], the question of what fraction of it is devoted to functional elements has attracted a considerable amount of interest and debate. Answering this question has been attempted using different empirical and computational approaches. One such approach is based on direct mapping of various elements in the human genome using assays that measure different types of biochemical events consistent with functionality, such as the interaction of a DNA binding protein with a specific DNA sequence or transcription of a particular DNA region. Using this approach, for example, it was discovered that mammalian genomes are pervasively transcribed to generate various classes of the so-called “RNA dark matter” transcripts of unknown function both inside and outside of the boundaries of protein-coding genes [2–5]. Furthermore, the identification of genomic elements using such a strategy has become the stated goal of the ENCODE [6, 7] and FANTOM [8] consortiums. In fact, >80% of the human genome sequence could be assigned a putative function using this strategy by the ENCODE consortium with as much as ¾ of the genome sequence used as the template for transcription [9]. However, detection of a biochemical event cannot per se be indicative of biological significance since no effect on cellular or organismal fitness can be inferred from such assays [10–12].

On the other hand, a different type of methodologies used various measures of sequence conservation in evolution to estimate the fraction of sequence that has some measurable effect on fitness [13]. Using such approaches, only a small fraction (<10%) of the human genome has been estimated to be resistant to evolutionary change [14]. However, such analyses require estimations of the background rates of neutral evolution, which in turn require making specific assumptions about genomic regions that have no biological relevance [15]. Typically, such regions are represented by ancient repeats, but there is a growing body of evidence that challenge this assumption by showing that many ancient repeats are functional and, therefore, cannot be used to estimate the fraction of evolutionarily constrained sequence [5]. Moreover, sequence conservation analyses would likely miss motifs that function at levels other than the primary sequence (for example, at the level of 2D or 3D RNA structure), functional sequences that recently emerged in evolution, or other functional elements — in fact, some functional long non-coding (lnc) RNAs do not exhibit sequence conservation [16–18]. The finding that regulatory sequences, such as promoters and lncRNAs, can evolve more rapidly that protein-coding sequences due to positive selection during adaptive radiation presents an additional level of complication to efforts aimed at the estimation of the fractions of functional sequences based on the evolutionary conservation-based strategies [5, 15, 16, 19, 20]. Furthermore, sequence conservation does not necessarily equal biological function since genetic knockouts of evolutionarily conserved sequences do not always result in phenotypes [21].

Therefore, other methodologies that can directly answer the question about biological functionality of many different genomic elements are required. In fact, a number of high-throughput reverse genetics assays that fulfill this need have been developed. Such assays are typically based on targeting multiple known genomic elements, for example, exons of known protein-coding genes [22, 23] or lncRNAs [24, 25], or their regulatory regions [26]. Such screens typically use RNAi [23, 27], antisense oligos [25], CRISPR/Cas9 family of methods [22, 24, 26, 28] or CRISPR/Cas13 [29]. Such approaches, while powerful, have two inherent prominent flaws. First, they typically target elements that have been previously identified, and are not well-suited for studying novel unannotated elements. Second, such screens are typically based on methods that use short targeting RNA (or DNA) molecules — for example, shRNA in RNAi or guide (g) RNA in the CRISPR-based suite of gene targeting technologies — that have well-known off-target and non-specific effects, at least in part due to partial sequence complementarity to non-targeted sequences in the complex genome [21]. Therefore, there is always an ambiguity as to the assignment of a phenotype to a genomic element detected in such screens. Thus, there is a strong need for alternative techniques in functional genomics that could overcome these limitations, or at the very least, provide independent validation of the functional elements derived from the reverse genetics approaches.

In this respect, functional screens based on the forward genetics insertional mutagenesis strategy do not rely on the existing knowledge of functional genomic elements and therefore are well suited to study uncharacterized genomic regions. Published studies that applied this strategy to mammalian cell lines were typically based on phenotypical analysis of libraries of cells containing exogenous viral genomes integrated into different locations. The effect of a viral insertion on cellular fitness could be determined by measuring dynamics of relative abundance of that insertion in cellular population. For example, insertions causing cells containing them to be depleted during growth would represent insertions with a negative effect on cellular fitness and those enriched — a positive effect. Precise mapping of cellular integration events allows for the connection of the integration sites to the phenotypes of interests and eventually to the genomic elements affected by the insertions. However, so far, such screens predominantly focused on the annotated genes as the sole class of genomic functional elements.

In this work, we established a lentiviral insertional mutagenesis method and demonstrated that such strategy, previously limited to very few haploid or near-haploid mammalian cell lines [30–33], can work in aneuploid human cell lines. Using this approach, we found that most (>70%) of insertions that can cause cellular phenotypes could not be explained by exons of annotated genes and their regulatory regions, thus arguing for presence of a very large number of unannotated functional elements in the human genome. We also show that some of such novel functional elements correspond to novel exons previously predicted only using in silico sequence analysis algorithm GENSCAN [34]. We demonstrated, using a combination of Rapid Amplification of cDNA Ends (RACE) and long-read Nanopore platform, that many of such in silico exons do in fact correspond to novel exons of novel genes or non-canonical isoforms of known genes. Moreover, while low abundant under normal conditions, these novel transcripts can be massively (up to 3,214-fold) induced by the stress. While the inclusion of novel exons can interrupt and truncate the canonical open reading frames (ORFs), we show that such transcripts can function as bi-cistronic mRNAs. Altogether, these results argue for widespread presence of yet unannotated exons of novel and known genes and for functionality of different types of transcripts, including non-canonical RNA species that resemble aberrant splice products of annotated genes.

## Results

### Establishment and validation of insertional mutagenesis screens in human aneuploid cell lines

**Fig. 1:**
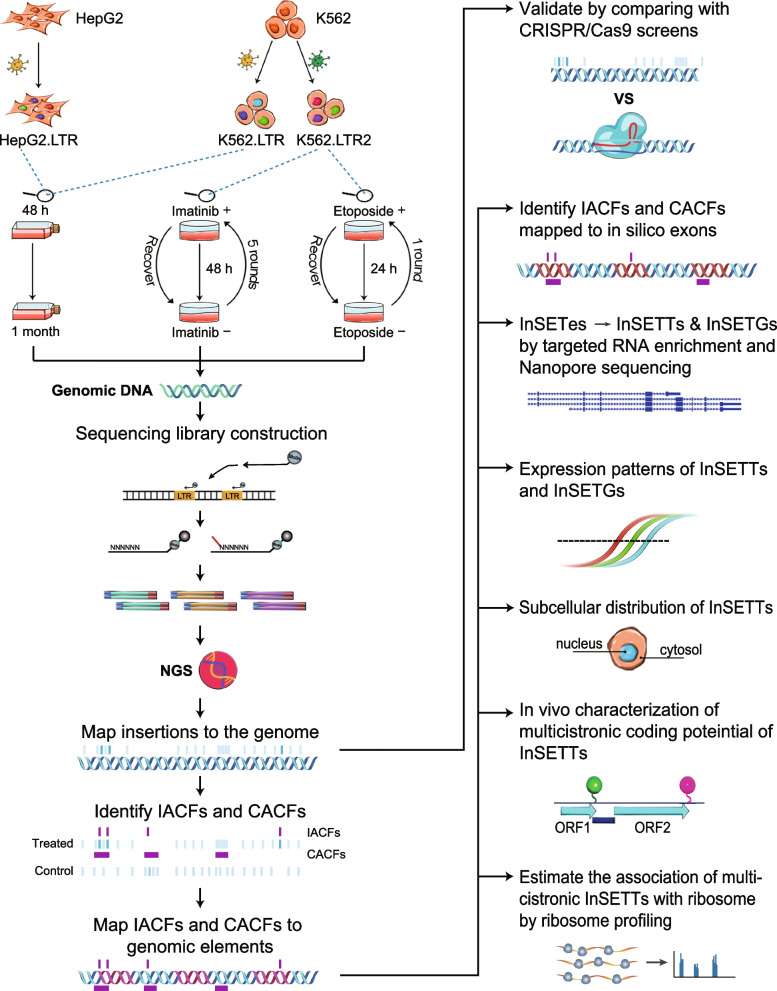
Flow chart diagram illustrating the steps of the project. The diagram depicts (1) biological systems used in this this study, (2) concept and validation of InSET method, and (3) discovery and characterization of novel transcripts and genes corresponding to the novel functional elements found by InSET method

As the first step in performing the lentiviral mutagenesis, we developed a biochemical and analytical pipeline to detect lentiviral insertions in the genome that we named InSET (*In*sertion-based *S*creen for functional *E*lements and *T*ranscripts) (Fig. 1, Additional file 1: Fig. S1, Methods). The biochemical basis of the method was similar to the FLEA-PCR [35] method and was further adapted for the NGS Illumina platform (Additional file 1: Fig. S1a and Methods). Then, we applied the pipeline to map insertions in 3 independently generated cell libraries — HepG2.LTR, K562.LTR, and K562.LTR2, each of which harbors lentivirus insertions of >5 kb in the genome that should disrupt the genomic elements (e.g., exons) in the insertion sites (Fig. 1 and Methods). In addition, due to the presence of enhancer/promoter sequences in the vectors required to drive expression of fluorescent proteins to allow for selection of transfected cells, certain amount of transactivation effect on nearby genes could be expected (see below).

The 3 libraries were used in 4 different phenotypic assay systems. The HepG2.LTR and K562.LTR cell lines were expanded for 2 days post transfection, after which the cells were split and, for each replicate, one aliquot was used for genomic DNA isolation and another aliquot was allowed to grow with normal frequency of passaging for 1 month and used for genomic DNA isolation. The K562.LTR2 cell line was allowed to expand for 3 months after the transfection and then subjected to survival experiment in response to two anticancer drug treatments essentially as described previously [29]. Briefly, cells were treated with 0.5 μM imatinib or 40 μM etoposide for 48 and 24 h respectively, then resuspended in fresh medium for recovery and passaged daily until most cells recovered the normal shape or the doubling rate of the untreated cells. Five and one rounds of drug treatment and recovery were performed for imatinib and etoposide respectively. The insertional mutagenesis screens in each phenotypic assay system were performed in 3 biological replicates.

As the first step, we evaluated the potential of lentiviral insertions to affect cellular fitness since only one copy of a functional element would be inactivated by an insertion that would cause relatively modest knockdown in the aneuploid cancer genome like the one present in K562 cells [36]. Indeed, inactivation-based insertional mutagenesis has so far been tested only in the very few mammalian haploid or near-haploid cell lines like KBM7 or HAP1 [30–33]. If insertions affect fitness, then cells harboring them would be lost, resulting in depletion of insertions in essential genes — a phenomena that has been observed in retroviral mutagenesis based on a haploid cell line [33]. To investigate whether this is indeed the case, we took advantage of the high-throughput CRISPR/Cas9 screen by Wang et al. aimed at identifying essential genes in K562 cell line [22]. Each gene in that study was assigned a CRISPR score (CS) defined by the authors as the average log_2_ fold change in the abundance of all single-guide (sg) RNAs targeting a given gene [22]. Negative CS score means depletion of cells expressing sgRNAs targeting specific gene and would thus signify importance of the gene for cellular fitness. Thus, such genes should be less likely to contain insertions.

**Fig. 2:**
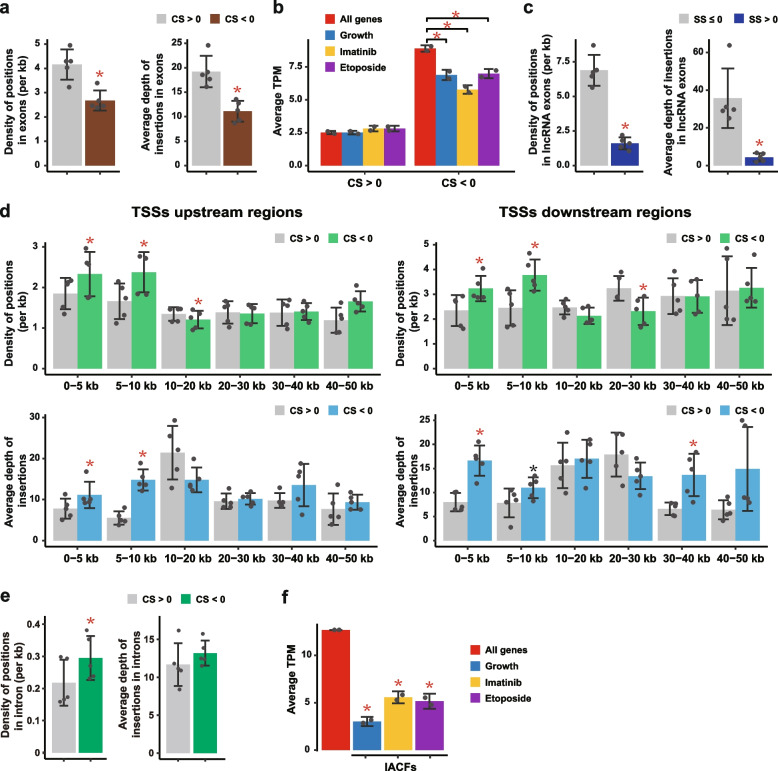
Validation of InSET method. **a** Density of positions and average depth of insertions in the exons of the CS > 0 and CS < 0 genes. **b** Average TPM of all CS > 0 or CS < 0 genes and of those with insertions in exons in the growth or drug survival challenge assays. **c**, **d** Density of positions and average depth of insertions in the exons of the SS ≤ 0 and SS > 0 lncRNAs (**c**) and in the various distance bins upstream or downstream of TSSs of the CS > 0 and CS < 0 genes (**d**). **e** Density of positions and average depth of insertions in the introns and not within ±10 kb region of TSSs of the CS > 0 and CS < 0 genes. **f** Average TPM of all genes and those with IACFs in exons. For average and median depth of insertions, insertion positions with insertion number greater than or equal to 10,000 were removed as outliers to avoid the bias. For density of positions and average depth of insertions, error bars indicate the SD based on 5 K562 sample types. For average TPM, error bars indicate SD of 2 biological replicates. Red asterisks indicate significant differences under two-sided paired Student’s *t* test (*p* value < 0.05). Black asterisk indicates *p* value = 0.05. Source data are provided in Additional file 2: Tables S1-S3

To test this, unique positions of insertions in the genome and number of insertions in each such position (depth) were determined for each sample and mapped to various types of genomic elements (Fig. 1 and Additional file 1: Fig. S1b). Since an insertion in an exon of a gene should destroy the function of that gene, we calculated density of insertions in exons of genes with CS < 0 and CS > 0 for every K562 sample based on unique positions of insertions (Fig. 2a and Additional file 2: Table S1). Indeed, for every sample, we observed a lower density of insertions in exons of the genes with CS < 0 compared to the genes with CS > 0 (Fig. 2a). This trend was statistically significant (*p* value of 1.4E−03, two-sided paired Student’s *t* test). We also observed lower average depth of insertions in the exons of genes with CS < 0 compared to depth in those with CS > 0 with the corresponding *p* value of 4.5E−03 (two-sided paired Student’s *t* test) (Fig. 2a and Additional file 2: Table S1). The reason for using both metrics (density of unique insertions and average depth) is that some insertion positions may have a very high depth and skew the results and using unique insertions would obviate this potential problem. On the other hand, the metric based on unique insertions could have lower sensitivity. Theoretically, true signal should have changes in both metrics.

An additional proof of the bias against the essential genes came from analysis of expression levels of all essential genes and those with insertions. As shown in Fig. 2b and Additional file 2: Table S2, the essential genes (CS < 0) with or without insertions had a strong tendency to have higher expression levels, as defined by transcripts per million (TPM) metrics measured by RNA-seq in untreated K562 cells, compared to the CS > 0 genes. Moreover, among the CS < 0 genes, the expression levels of genes with insertions were significantly lower than all genes with CS < 0 (*p* value < 0.05, two-sided paired Student’s *t* test) (Fig. 2b and Additional file 2: Table S2). These observations suggest that cells harboring insertions in higher expressed essential genes have lower survivability and thus are preferentially lost in the screens.

We then explored whether insertional mutagenesis can affect cellular fitness via inactivation of functional genomic elements other than exons of protein-coding genes. Since the annotated genes tested in the above study by Wang et al. [22] are mostly represented by canonical protein-coding mRNAs, we further tested effects of insertions in lncRNAs using a similar approach. The study by Liu et al. has conducted a genome-wide CRISPR/Cas9 screen for functional lncRNAs in K562 cell line and generated a screen score (SS) for every lncRNA transcript [37]. A lncRNA with a higher SS was considered to be more essential [37]. Similar to the results in the annotated genes above, the density and average depth of insertions in the exons of lncRNAs with SS > 0 were significantly lower than the corresponding values for the lncRNAs with SS ≤ 0, with the corresponding *p* values of 9.0E−04 and 8.2E−03 (two-sided paired Student’s *t* test) (Fig. 2c and Additional file 2: Table S3). Altogether, these results strongly suggest that detection of insertions is biased against more essential genes and lncRNAs, most likely because cells containing those insertions do not survive even after a very short time (2 days) in culture after lentivirus integration. These observations are consistent with the previous results obtained with a haploid cell line [33] and suggest that inactivation of one copy of a genomic element, such as an exon, via lentiviral insertion in an aneuploid cell line can have a measurable effect on fitness, and therefore insertional mutagenesis can reveal biologically relevant genomic elements in such system.

An inherent feature of insertional mutagenesis strategies based on retroviral insertions is that the insertions can sometimes activate expression of nearby genes via enhancer/promoter sequences present in the viral genomes [38, 39]. Upregulation of essential genes due to insertion of lentivirus in the vicinity of their transcription start sites (TSSs) could theoretically increase cellular fitness. To test whether this is indeed the case in our system, we estimated density and average depths of insertions in various bins of distances upstream or downstream from annotated TSSs for the genes with CS < 0 and CS > 0. To calculate the insertions in the regions downstream from TSSs, insertions in exons were not counted. The 0-5 kb and 5-10 kb upstream and downstream bins were the only 4 bins where most consistent statistically significant difference in both the density and average depths of insertions between the two groups of genes were observed (Fig. 2d and Additional file 2: Table S1). Also, in both distance bins, the density and average depths were higher in the CS < 0 group of genes (Fig. 2d and Additional file 2: Table S1). Therefore, these results are consistent with the notion that in addition to disruption of a genomic element, the effect of activation of nearby genes could also exist. However, this effect appears to be limited to insertions located within ±10 kb region of nearby TSSs. Furthermore, the effect of insertion-caused depletion was stronger than that of the transactivation according to the Hedge’s *g* estimators of the sizes of these two types of effects. Specifically, the absolute Hedge’s *g* value for the density of positions in exons of essential vs non-essential genes was 2.53 while the average Hedge’s *g* value for the 4 distance bins in ±10 kb region around TSSs was 1.37 (Additional file 2: Table S1). The corresponding values for the average depth of insertions were 2.69 and 2.26 respectively (Additional file 2: Table S1).

Conceivably, insertion of a retrovirus insertion into an intron of a gene could indirectly affect the function of that gene, for example by providing alternative splice sites or transcription termination signals. To estimate whether this could be a common phenomenon, we further calculated the density of insertion positions and depth of insertions in introns and not within ±10 kb region of TSSs in CS < 0 and CS > 0 genes. As shown in Fig. 2e and Additional file 2: Table S1, there was no bias against integrations into the introns of essential (CS < 0) genes. In fact, a significantly higher density of positions, but not depth of insertions, could be observed in CS < 0 genes. Therefore, in contrast to significant and consistent deleterious effect of insertions in exons (Fig. 2a, b), these results suggest that integration into introns do not have a widespread disrupting effect on the corresponding genes.

Taken together, these results suggest that the most common phenotypic effect of a lentiviral insertion, at least in our system, is mediated by direct disruption of the functional element, such as an exon of a spliced transcript, targeted by the insertions. The presence of some transactivation effect should also be considered if the insertions happen to appear within ±10 kb region of the TSSs of nearby genes. In the following part, we will focus on the genomic elements harboring insertions as the candidates for downstream analyses unless stated otherwise.

### InSET reveals that most of sequences affecting cellular fitness map outside of annotated genomic elements

To detect insertions that affect functional genomic elements, we identified insertion sites that affect fitness in various phenotypic assay systems used in this study. Unique sites enriched or depleted during normal growth of the HepG2.LTR and K562.LTR cell lines were obtained by comparing depth of insertions of each unique position after 1 month with that at 2 days as control (Fig. 1). Unique sites enriched or depleted following the anticancer drug survival experiment in the K562.LTR2 cell line were obtained by comparing the depth of each unique position in cells after the survival challenge to that in cells before the challenge (Fig. 1). The genomes of cells after growth and drug treatment should be the same as those of the control cells since they were based on the same initial population of transfected cells. Therefore, we do not expect that depletion or enrichment of insertions to be caused by the copy number variation. As summarized in Additional file 2: Table S4, we could detect 3,574,490 sites of insertions across the 4 phenotypic assay systems where the insertion sites in the same genomic location but detected in different systems were counted as independent insertions, of which 2,845,600 (79.6%) were unique insertion sites. Using 25% false discovery rate (FDR) threshold, 29,366/3,574,490 (0.8%) individual sites representing insertions affecting cellular fitness (IACFs) could be detected as either depleted or enriched in at least one system (Additional file 2: Table S4). Among the IACFs, 28,987 (98.7%) were unique. Genes harboring IACFs in exons had consistently lower expression levels than all genes (Fig. 2f and Additional file 2: Table S2), consistent with the results above showing that our system is biased against detecting essential genomic elements.

**Fig. 3:**
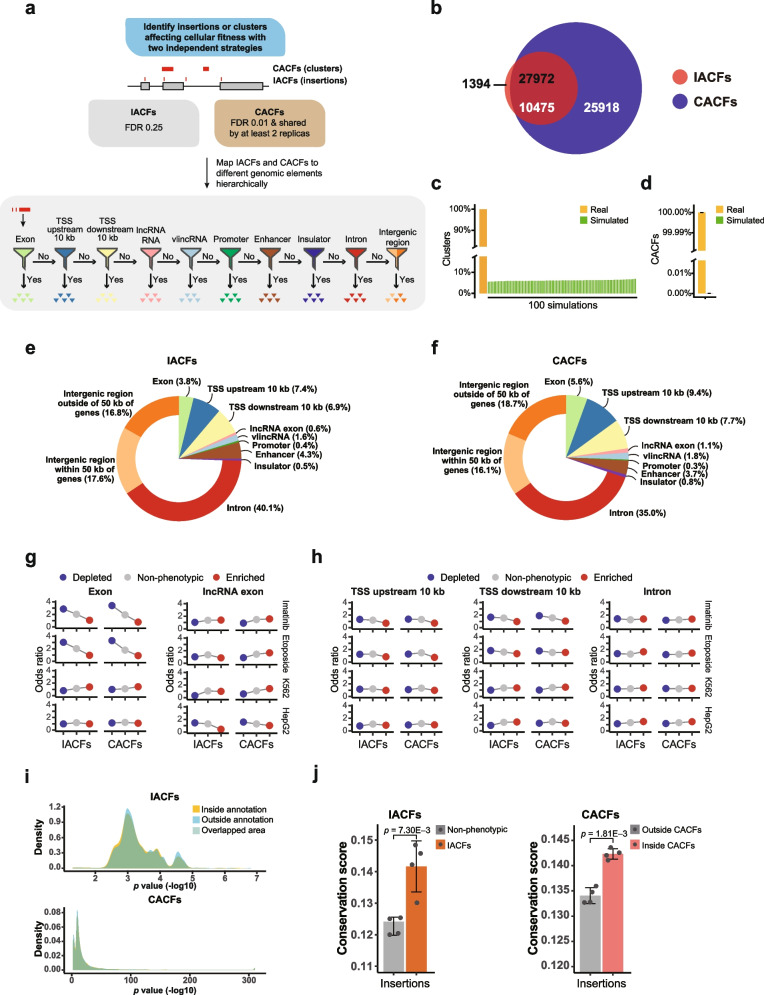
Genome-wide distribution of IACFs and CACFs. **a** Schematic diagram of the analytical pipeline for identification and hierarchical mapping of IACFs and CACFs to different genomic elements and regions shown in the pie charts in the panels **e** and **f**. Number of insertions in each site was compared among the 3 biological replicates of the samples subjected to a phenotypic challenge (time of growth or drug treatment) and the corresponding control to identify IACFs using FDR threshold of 25%. In the independent approach, clusters of phenotypic insertions were first identified using SICER with a FDR threshold of 1%, and those shared by at least 2 biological replicates were defined as CACFs. For more details, see Additional file 1: Fig. S1. **b** Venn diagram of overlap between IACFs (black) and CACFs (white). The numbers represent non-unique events where the count for the same insertion or cluster would be added for each system where it was found. **c**, **d** Ratio of real vs simulated SICER clusters (**c**) and CACFs (**d**) based on 100 simulations for one representative example of the simulation analysis for insertions depleted in one biological replicate of the K562 growth survival experiment (see Additional file 1: Fig. S2 for the complete set of simulation analyses). Error bars indicate SD. **e**, **f** Genome-wide distributions of all IACFs and CACFs across the 4 phenotypic assay systems. Each unique IACF and CACF position was assigned to only one genomic element or region as shown in **a**. **g**, **h** Odds ratios of enrichment of the phenotypic (depleted or enriched) and no phenotype insertions in different genomic elements.** i** Distribution of *p* values of IACFs and CACFs mapping inside or outside of the annotations defined as all elements from exons to insulators in panel **a**. **j** Conservation scores of the phenotypic vs non-phenotypic insertions based on IACFs (left) or insertions located inside or outside of the CACFs (right). Error bars indicate SD based on the 4 phenotypic assay systems. The *p* values were calculated with one-sided paired Student’s *t* test. Source data are provided in Additional file 2: Tables S4-S10

However, the analysis based on single insertion events would suffer from lower sensitivity and potentially higher false positive rate. Therefore, we used an independent analytical approach based on the assumption that true functional elements should be detected by multiple independent phenotypic insertion events. Such clusters were calculated in a two-step process. First, we used SICER software [40] with a very stringent 1% FDR threshold to identify genome-wide clusters of nearby insertion events that were either enriched or depleted in each of the 3 pairs of biological replicates of each phenotypic assay system (Figs. 1 and 3a, Additional file 1: Fig. S1, Methods). The input into the SICER program were the positions of the raw insertion events in each biological replicate of treatment with either 3-month culture or drug challenge and in the corresponding control biological replicate. Second, clusters shared by at least 2 out of 3 biological replicates were defined as clusters affecting cellular fitness (CACFs) (Fig. 1, Additional file 1: Fig. S1, Methods). We could detect 36,393 CACFs across the 4 phenotypic assay systems. The CACF analysis was based on raw insertions and therefore independent of IACF; however, most (27,972/29,366 or 95.3%) IACFs were found inside CACFs. On the other hand, since analysis based on multiple insertions would be expected to be more sensitive, many (25,918/36,393 or 71.2%) CACFs did not contain IACFs (Fig. 3b). CACFs with overlapping coordinates or detected in different systems were counted independently. However, merging coordinates of the 36,393 CACFs resulted in 18,531 (50.9%) unique CACFs, suggesting that the functional clusters were much more common to different systems than individual IACFs.

To validate the procedure of CACF detection, we performed a two-step simulation analysis as follows. For each sample, we generated the same number of insertions with random coordinates across the genome and used SICER to generate clusters. After 100 simulations, the maximum number of simulated clusters varied between 2.2 and 10.6% of the number of the real clusters for the different types of samples (Fig. 3c, Additional file 1: Fig. S2a, Additional file 2: Table S5). These results shows that SICER, originally designed to identify regions enriched in ChIP-seq analysis [40], can indeed identify truely enriched or depleted clusters of insertions in the lentiviral mutagenesis context. Second, when the simulated clusters shared by at least two replicates were used to generate simulated CACFs, virtually no CACFs were obtained: the average ratio of simulated CACFs compared to the real ones varied between 0 and 7 × 10^−6^ (Fig. 3d, Additional file 1: Fig. S2b, Additional file 2: Table S6). Overall, these results clearly show that CACFs were not random.

We then investigated genomic distribution of IACFs and CACFs (Fig. 3a and Additional file 2: Tables S4 and S7). A small fraction of IACFs and CACFs mapped to exons of known genes (3.8 and 5.6%, Fig. 3e, f), and comparable numbers of IACFs and CACFs (0.6 and 1.1% respectively) mapped to exons of annotated lncRNAs. Furthermore, additional 1.6 and 1.8% of IACFs and CACFs mapped to very long intergenic non-coding RNAs (vlincRNAs) [41] (Fig. 3e, f). We then explored contribution of potentially functional elements defined by potential regulatory elements detected based on DNA-protein interactions. Additional 0.4% and 0.3%, 4.3% and 3.7%, and 0.5% and 0.8% of IACFs and CACFs mapped to promoters, enhancers, or insulators found in K562 and HepG2 cells based on ChIP-seq mapping of chromatin states [42, 43] and located outside of the above mentioned elements (Fig. 3e, f).

We then calculated odds ratios of enrichment of IACFs and CACFs in the annotated functional elements to further characterize and validate performance of the method. Interestingly, the strongest enrichments were observed for depleted IACFs and CACFs found in exons of protein-coding genes, but only in the phenotypic assay systems where cells were challenged with the anticancer drugs (Fig. 3g and Additional file 2: Tables S8 and S9). This is most likely due to the fact that insertions in exons of genes required for growth have already been removed from the cellular population as has been shown above. However, new survival stress to which cells have not yet been exposed, like the one provided by the anticancer drug treatments, revealed additional genes required for stress resistance. These results also illustrate the limitation of the method — insertions in the elements required for growth and survival under normal conditions would be under-represented in insertional mutagenesis screens.

On the other hand, our results strongly argue that most of the phenotypic insertions do not exert their effect by somehow affecting protein-coding genes. As can be seen on the Fig. 3h and Additional file 2: Tables S8 and S9, depleted or enriched insertions were not significantly more enriched in the immediate vicinity of the TSSs of genes or inside the introns. However, if the phenotypic insertions identified in our screens did in fact function by somehow affecting functions of known genes, either by transactivation, or by affecting splicing or terminating transcription, then the opposite would be expected. In fact, strikingly, the vast majority (96.2 and 94.4%) of IACFs and CACFs were located outside of exons of known genes (Fig. 3e, f and Additional file 2: Tables S4 and S7). Even considering possible transactivating effects, most (81.9% and 77.3%) of IACFs and CACFs were located outside of exons of known genes, or 10 kb up- or downstream from their TSSs (Fig. 3e, f and Additional file 2: Tables S4 and S7). Moreover, we did not observe increase in enrichment of phenotypic insertions in annotated exons of lncRNAs (Fig. 3g and Additional file 2: Tables S8 and S9), arguing that the annotated spliced lncRNAs cannot explain most of the phenotypic insertions observed in this work, with the caveat though that many lncRNAs and their exons are not present in the current genomic annotations (see “ Discussion”). Even accounting for all of the annotated functional elements (exons of lncRNAs, regulatory elements, and so on), 69.8% and 74.5% of IACFs and CACFs did not map to any functional or potentially functional genomic region (Fig. 3e, f and Additional file 2: Tables S4 and S7). Furthermore, 34.4 and 34.8% of IACFs and CACFs were found in the intergenic space and outside of any functional or potentially functional genomic region (Fig. 3e, f and Additional file 2: Tables S4 and S7). Moreover, 16.8 and 18.7% of IACFs and CACFs mapped to “gene desert” regions, 50 kb from boundaries of known genes (Fig. 3e, f and Additional file 2: Tables S4 and S7). To estimate whether IACFs and CACFs mapping outside of the known functional or potentially functional elements were somehow biased to being less significant compared to those mapping to these annotations, we compared the *p* value distribution of the two groups of IACFs and CACFs, and found that the distribution of the *p* values of IACFs and CACFs mapping inside and outside of the annotated elements matched well (Fig. 3i). Taken together, these results show that the human genome is likely to contain multiple yet uncharacterized elements that have physiological significance.

To validate potential biological relevance of the novel elements tagged by the phenotypic insertions, we asked whether such insertions correspond to more evolutionarily conserved sequences compared to the insertions that did not result in phenotypes. In this analysis, insertions located in the annotated exons or within 10 kb upstream/downstream of TSSs of known genes were excluded. Interestingly, the evolutionary sequence conservation, as defined by the phastCons conservation scores of the genomic base pairs immediately adjacent to the integration sites (Methods), was significantly higher for the IACFs than insertions with no phenotypes, and for the insertions located inside CACFs than those located outside (Fig. 3j and Additional file 2: Table S10). Overall, these results argue that the phenotypic insertions detected in this work do indeed mark some unknown functional elements in the human genome.

### IACFs or CACFs mapping to unannotated genomic regions can disrupt novel exons

IACFs or CACFs mapping outside of genomic regions of known functions could potentially affect multiple types of functional genomic elements, requiring a plethora of different types of assays to uncover their mechanisms of action. However, based on the analysis of the insertions mapping to exons of essential genes and lncRNAs shown above, it is reasonable to assume that IACFs or CACFs could exert their effects by disrupting exons of novel transcripts. Therefore, to prove that IACFs or CACFs that mapped to unannotated genomic space could potentially affect novel bona fide genomic elements, we focused on such IACFs or CACFs that mapped to exons predicted using in silico sequence analysis program GENSCAN [34] (Additional file 2: Tables S8 and S9). It is important to stress however that GENSCAN program was designed based on the general transcriptional, splicing and translational signals of protein-coding genes [34]; therefore, the corresponding predictions are biased towards sequences with protein-coding potential.

**Fig. 4:**
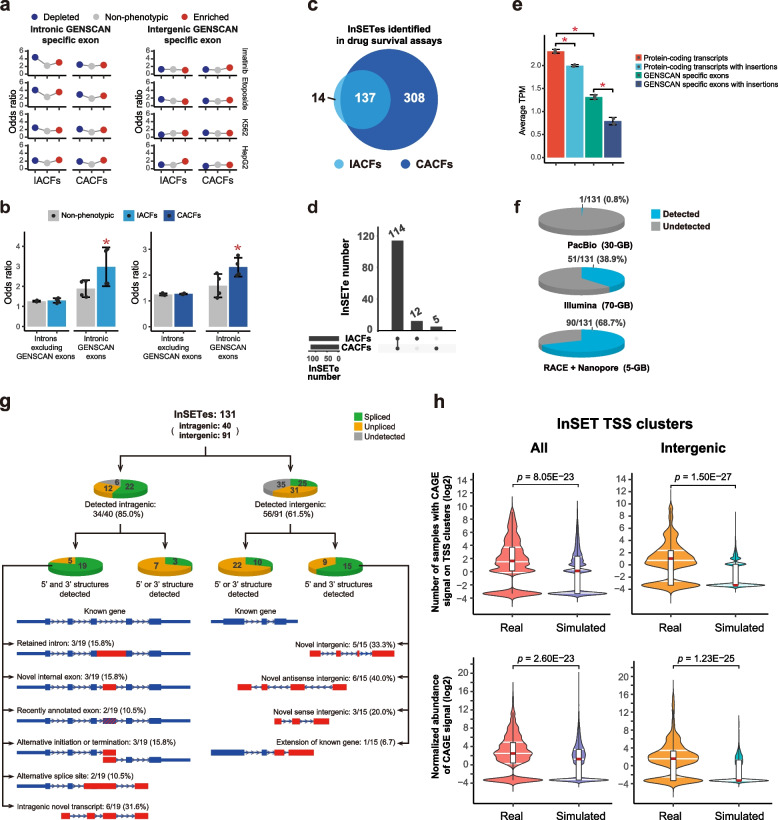
Characterization of InSETT and InSETG structures by Nanopore sequencing. **a** Odds ratios of enrichment of the phenotypic (depleted or enriched) and non-phenotypic insertions in the intronic and intergenic GENSCAN-specific exons. **b** Odds ratios of enrichment of IACFs, insertions in CACFs and non-phenotypic insertions in the intronic GENSCAN-specific exons or in intronic regions outside of the GENSCAN-specific exons. Error bars indicate SD based on the 4 systems (K562 growth, HepG2 growth, imatinib survival challenge, and etoposide survival challenge). Asterisks indicate significant differences per two-sided paired Student’s *t* test (*p* value < 0.05). **c**, **d** Overlaps of InSETes detected by the IACF and CACF analysis in the K562 drug survival assays genome-wide (**c**) and among the 131 exons selected for the RACE assays (**d**). **e** Average TPM of all protein-coding transcripts and those with insertions in their exons, and of all GENSCAN-specific exons and those with insertions. Error bars indicate SD based on 2 biological replicates. Asterisks indicate significant differences under two-sided paired Student’s *t* test (*p* value < 0.05). **f** Sensitivity of detection of novel functional transcripts with PacBio, Illumina, or with the RACE enrichment followed by Nanopore sequencing. Note: the relatively short Illumina reads overlapping InSETes could be derived from transcripts different from those detected by RACE/Nanopore. **g** Summary of the types of novel transcripts detected by the RACE combined with Nanopore sequencing for the 131 InSETes. Transcript classification was done based on RACE, unless only RT-PCR results were available. **h** CAGE signal in the real and simulated InSET TSS clusters. For each InSET TSS cluster (left) or intergenic InSET TSS cluster (right), the number of tissue or primary cell samples with overlapping CAGE tags (upper) and the normalized abundance of the CAGE signal (lower) were calculated. In the boxplots, center red lines indicate median; box limits indicate upper and lower quartiles; whiskers extend from the box limits no more than 1.5× interquartile range. In the violin plots, all violins were scaled to have the same maximum width; three horizontal white lines indicate upper, median, and lower quartiles of the density estimate respectively. All data including TSS clusters with 0 CAGE tags were plotted. To plot on the log scale, 0.1 was added to all values. The *p* values were calculated using Wilcoxon rank-sum test. Source data are provided in Additional file 2: Tables S8, S9, and S11-14

Strikingly, the phenotypic insertions, both depleted and enriched, were consistently enriched in exons found solely by GENSCAN program and not present in the databases of annotated human genes such as UCSC Genes [44] or GENCODE [45, 46] — the GENSCAN-specific exons (Fig. 4a and Additional file 2: Tables S8 and S9). Importantly, the enrichment was higher than that of the insertions with no phenotypes in any phenotypic assay system tested (Fig. 4a and Additional file 2: Tables S8 and S9). Interestingly, however, the increase in the enrichment of phenotypic insertions was limited to GENSCAN-specific exons, found in introns of known genes, as opposed to those found in the intergenic space (Fig. 4a and Additional file 2: Tables S8 and S9). Also, there was no increase in the enrichment of phenotypic insertions in the intronic regions outside of the GENSCAN-specific exons (Fig. 4b). This strongly suggests that phenotypes caused by insertions into intronic GENSCAN-specific exons were caused by direct insertions in these exons and not by indirect disruption of the host gene.

The results presented above strongly argue that GENSCAN-specific exons, especially those of annotated genes, represent novel functional elements. Since such exons were identified solely based on in silico sequence analysis and were not present in the current human gene annotations, we first focused on identification of transcripts corresponding to GENSAN-specific exons harboring phenotypic insertions found in any of the 4 phenotypic assay systems. Overall, 4.5% (1,320/29,366) of IACFs and 4.5% (1,651/36,393) of CACFs mapped to exons predicted only by GENSCAN. In the drug survival experiment, 151 and 445 such GENSCAN exons harbored IACFs and CACFs respectively, among which 137 harbored both IACFs and CACFs (Fig. 4c). For this analysis, we have chosen 131 GENSCAN exons harboring IACFs and/or CACFs (114 exons contained both (Fig. 4d)) that will be referred to as InSET exons or InSETes. The 131 InSETes consist of 40 intragenic exons defined as being inside the known genes and on the same strand as the genes, and 91 intergenic exons defined as being outside or antisense to known genes (Additional file 2: Table S11).

We initially tested the overall abundance of all GENSCAN-specific exons in the human genome and the 131 InSETes by RNA-seq analysis using high-throughput short-read Illumina platform. We found that while GENSCAN-specific exons could be detected, their average abundance (TPM = 1.31) was significantly lower than that of protein-coding transcripts (TPM = 2.31, *p* = 0.041, two-sided paired Student’s *t* test, Fig. 4e), potentially explaining why they remain largely unannotated. Furthermore, similar to the exons of known genes, GENSCAN-specific exons with insertions had even lower abundance than those without: the corresponding average TPM of 0.79 vs 1.31 (*p* = 0.028, two-sided paired Student’s *t* test, Fig. 4e), suggesting that the insertions of in silico predicted exons important for cell survival were also subjected to loss. Nonetheless, the RNA-seq analysis suggested that the exons predicted only in silico can in fact represent bona fide exons. In fact, 51 or 38.9% of the 131 InSETes could be detected with the coverage of over 0.5 based on Illumina deep RNA-seq (Fig. 4f). However, the relatively short reads were not sufficient to define the isoforms of the corresponding transcripts; therefore, we performed long-read NGS analysis using the PacBio platform. However, after analysis of 656,065 circular consensus sequencing (CCS) reads obtained in this experiment (average read length 3038 nt), we could identify 1 CCS read corresponding to only 1 out of 131 such exons (0.8%) (Fig. 4f). Therefore, long-read RNA-seq analysis of a complex RNA population lacked the sensitivity to detect transcripts containing such exons.

Thus, as the next step, we tried two targeted enrichment strategies in combination with the long-read NGS Nanopore sequencing to identify such transcripts using the same wild-type non-transfected K562 RNA sample as in the PacBio RNA-seq analysis. First, for the 40 intragenic InSETes, we directly tested connection between the predicted exons and neighboring annotated exons using RT-PCR and found connection for 20/40 InSETes. Second, for all 131 intragenic and intergenic InSETes, we used 5′ and 3′ RACE to determine full-length transcript structure. Previous application of targeted transcript enrichment using 5′/3′ RACE in conjunction with tiling arrays has demonstrated a highly sensitive nature of this approach for discovery of novel transcripts and genes [47–49]. The combination of RACE with the long-read NGS platform combined the sensitivity of targeted transcript enrichment with the ability to directly obtain the full-length sequences of the RACE products, and thus allowed to reconstruct the underlying transcripts. This approach revealed presence of transcripts for 90/131 InSETes as evidenced by at least one (5′ or 3′) RACE reaction (Fig. 4f, g).

Overall, using both strategies we could detect presence of transcripts for 90/131 (68.7%) InSETes with 34/40 and 56/91 representing exons inside and outside of the gene boundaries respectively (Fig. 4f, g). Two of the 40 intragenic exons were found in the more updated versions of the UCSC Genes and GENCODE databases (Fig. 4g). The sequences of exons obtained from our RACE analysis matched those of the more recently annotated exons thus providing additional validation for the RACE approach as exemplified in Additional file 1: Fig. S3.

Interestingly, 22/34 (64.7%) and 25/56 (44.6%) of respectively detected intra- and intergenic InSETes corresponded to spliced transcripts (Fig. 4g). To further explore the structures of the corresponding transcripts, we focused on 48 InSETes (24 intragenic and 24 intergenic) where we could get positive RACE and/or RT-PCR products in both 5′ and 3′directions, thus providing more complete annotation of structures of the corresponding transcripts (Fig. 4g and Additional file 2: Tables S12 and S13). Of those, 19/24 (79.2%) intragenic and 15/24 (62.5%) intergenic exons corresponded to spliced transcripts (Fig. 4g). As shown below, some of the latter represent apparent novel isoforms of known genes while some represent novel genes. Below, we will refer to them as respectively InSETTs and InSETGs.

**Fig. 5:**
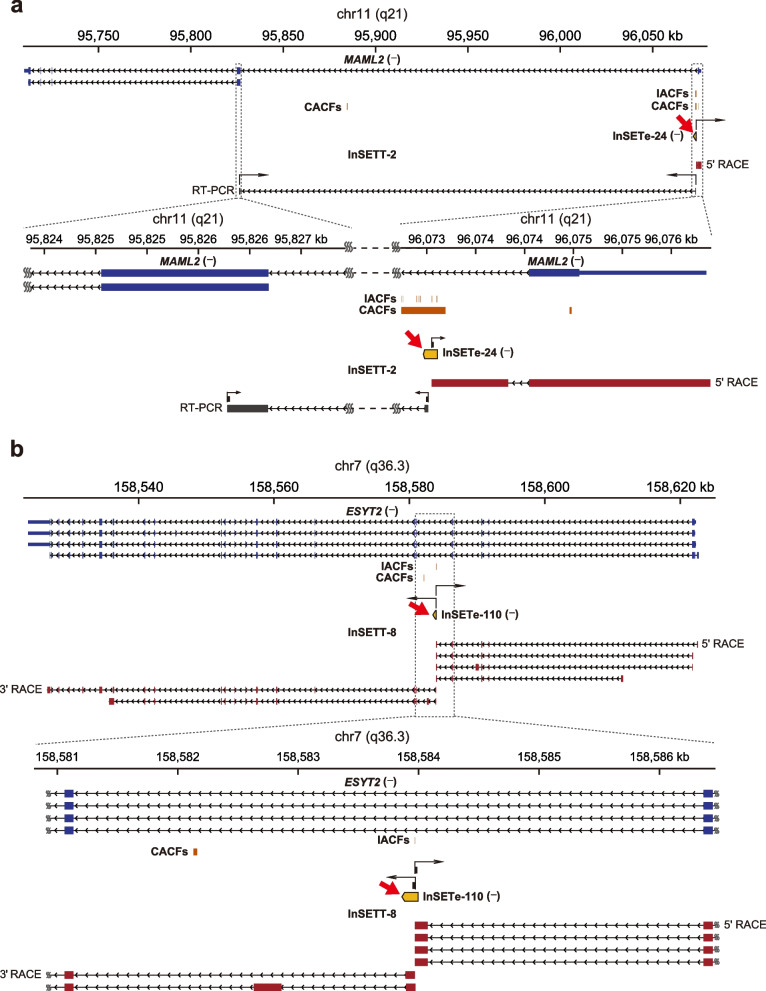
Examples of structures of novel intragenic transcripts. Nanopore sequencing results of products of 5′/3′ RACE or RT-PCR for InSETe-24 and InSETe-110 inside correspondingly *MAML2* (**a**) and *ESYT2* (**b**) genes. The bottom portions represent zoom-in views of the boxed regions above. For *ESYT2*, only representative transcripts are shown: for full depiction of 5′/3′ RACE results see Additional file 1: Fig. S5. Known genes annotations are in blue and based on the GENCODE Genes track from the UCSC Genome Browser. The InSETes are in yellow and marked by the red arrows. Structures of novel transcript InSETT-2 or the InSETT-8 cluster of novel transcripts (sharing InSETe-110) found in correspondingly *MAML2* and *ESYT2* loci by 5′/3′ RACE (red) and RT-PCR (black) are shown. IACFs and CACFs are in brown and represent unique insertions and merged clusters from only the drug survival system. Black arrows indicate the RACE or RT-PCR primers

As shown in Fig. 4g, of the 19 intragenic exons corresponding to spliced transcripts, 3 (15.8%) corresponded to events where the entire intron containing an InSETe was retained as illustrated for the exon found in an intron of *TPCN2* gene (Additional file 1: Fig. S4). On the other hand, 3 (15.8%) of such exons corresponded to novel internal cassette exons of known genes as illustrated on Fig. 5 and Additional file 1: Fig. S5 for the *MAML2* and *ESYT2* genes and Additional file 1: Fig. S6a for the *DNAH8* gene. Three out of 19 (15.8%) intragenic exons corresponded to alternative initiation or termination events as exemplified by the InSETes in the *IQSEC1* and *EHD1* genes (Additional file 1: Fig. S7a, b). Two out of 19 (10.5%) corresponded to exons with alternative splice site usage as exemplified by InSETes in *C1RL* and *SLC24A1* genes (Additional file 1: Fig. S7c, d). Finally, 6/19 (31.6%) of intragenic exons corresponded to novel transcripts on the same strand as the annotated genes, but apparently not being isoforms of these genes as exemplified by the InSETT-5 cluster of transcripts sharing the same InSETe in the intron of *COL18A1* and *PVT1* genes (Additional file 1: Fig. S7e, f).

**Fig. 6:**
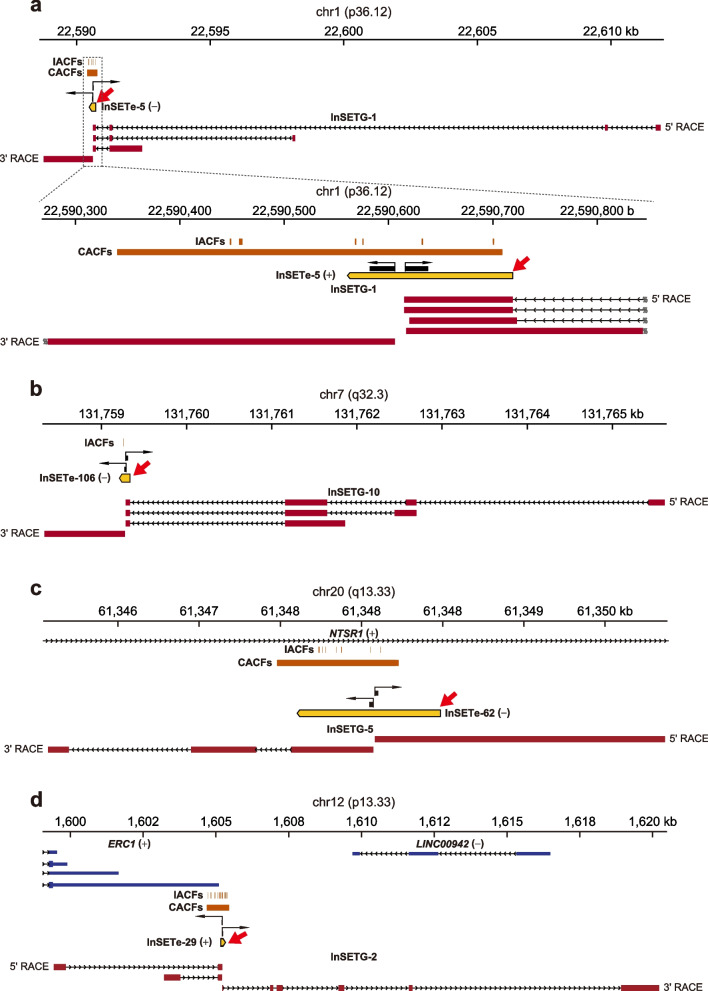
Examples of structures of novel intergenic transcripts. Nanopore sequencing results of 5′ and 3′ RACE for intergenic exons InSETe-5 (**a**), InSETe-106 (**b**), InSETe-62 (**c**), and InSETe-29 (**d**). Known gene annotations are in blue and based on the GENCODE Genes track from the UCSC Genome Browser. The InSETes are in yellow and marked by the red arrows. Transcript structures obtained from 5′/3′ RACE are shown in red. IACFs and CACFs are in brown and represent unique insertions and merged clusters from only the drug survival system. Black arrows indicate the RACE primers

Of the 15 intergenic InSETes corresponding to the spliced transcripts, only one represented a novel 3′extension of a known protein-coding gene (Fig. 4g and Additional file 1: Fig. S8). Thus, 14 out of the 15 InSETes apparently represented novel human genes found in this study. Five out of the 15 (33.3%) were located exclusively in the intergenic space as illustrated by the genes InSETG-1 and InSETG-10 (Fig. 6a, b). On the other hand, 6/15 (40%) and 3/15 (20%) InSETes overlapped known genes either on the opposite (antisense) or same (sense) strands and thus also corresponded to novel genes (Fig. 4g). Antisense InSETG-5 was located in an intron of *NTSR1* gene (Fig. 6c). Sense InSETG-2 overlapped 3′ UTR of *ERC1* gene on the same strand, but represented an apparently independent transcript unit (Fig. 6d). All in all, these results show that majority of the InSETes — either inside or outside known gene boundaries — represent novel transcripts or genes, and majority (34/48, 70.8%) of these transcripts are spliced (Fig. 4g).

In many examples above, RACE analysis also revealed additional novel exons connected to the original InSETe and not found in annotations as exemplified by *ESYT2* locus (Fig. 5b and Additional file 1: Fig. S5) and InSETGs shown in Fig. 6. Interestingly, in the case of *ESYT2* locus, two such exons also contained a CACF (Additional file 1: Fig. S5) and were not predicted by GENSCAN, suggesting that IACFs and CACFs found in this analysis that do not overlap known or predicted exons can still affect yet un-discovered transcripts.

To directly prove the expression of novel transcripts identified in this work in normal human tissues or cells and also test the validity of the 5′ RACE approach in identification of true TSSs, we took advantage of the Cap Analysis of Gene Expression (CAGE) dataset generated by the FANTOM 5 Consortium from 188 normal human tissue and 512 primary cell samples [8]. For this analysis we used the 5′ ends of 23 InSETGs, 25 InSETTs and transcripts of 30 InSETes for which 5′ RACE results were available. Since CAGE tags specifically mark positions of 5′ ends of transcripts, we tested the presence of CAGE tags in the immediate vicinity (±10 bp and on the same strand) of the 5′ ends of the novel transcripts identified using 5′ RACE. Prior to the analysis, we removed the 5′ ends of the novel transcripts that were in the immediate vicinity (±10 bp and on the same strand) of the annotated TSSs of protein-coding mRNAs to ensure that the results are not confounded by the signal from TSSs shared by known and novel transcripts. Overall, we could detect expression of 87.0% (20/23) InSETGs, 100% (25/25) InSETTs and transcripts of 80.0% (24/30) InSETes using this approach in at least one sample (Additional file 2: Table S14). However, many novel transcripts were detected in multiple samples, for example, of the 78 novel transcripts tested, 51 (65.4%) were detected in 5 or more samples.

To test whether the overlap with the CAGE tags occurred by random chance, we performed a simulation analysis for which we extended the novel TSSs by ±10 bp on the same strand and then merged them into 639 TSS clusters. We then generated 12,780 simulated TSS clusters that had the same fractions mapping to various genomic regions — exons, introns, antisense intragenic and intergenic — as the real TSS clusters (Additional file 2: Table S15). We also analyzed the intergenic fraction of the simulated TSS clusters independently. In the real data, 78.6 and 72.8% of all TSS clusters and intergenic TSS clusters respectively had overlapping CAGE tags in any of the human tissue or primary cell samples, while corresponding ratio in the simulated data is 63.7 and 41.6% (Additional file 2: Table S16), both of which were significantly lower than the ratio in the real data (*p* < 1 × 10^5^, chi-square test).

For each real and simulated TSS cluster, we further calculated the number of tissue or primary cell samples with overlapping CAGE tags, as well as the sum of normalized CAGE abundance in these samples. The real data had significantly higher CAGE signal than in the simulated data, with regard to either the number of samples with the overlapping CAGE tags or the normalized abundance of the CAGE signal (Fig. 4h and Additional file 2: Table S17). This was true for both all and only intergenic TSS clusters (Fig. 4h and Additional file 2: Table S17). Interestingly, the difference between the real and simulated data in the intergenic TSS clusters was more obvious than in all TSS clusters (Fig. 4h and Additional file 2: Table S17), reflecting a higher background CAGE signal in annotated genes. Thus, the CAGE signal from the intergenic regions could better reflect the expression of novel transcripts. However, it is worth mentioning that even in the intragenic region, significant difference between real and simulated data was observed, suggesting the novel transcripts from the intragenic regions are also expressed. Taken together, these results support the existence of novel transcripts in normal cells.

### Novel transcripts harboring IACFs or CACFs identified in the drug survival screens are induced by the drugs

**Fig. 7:**
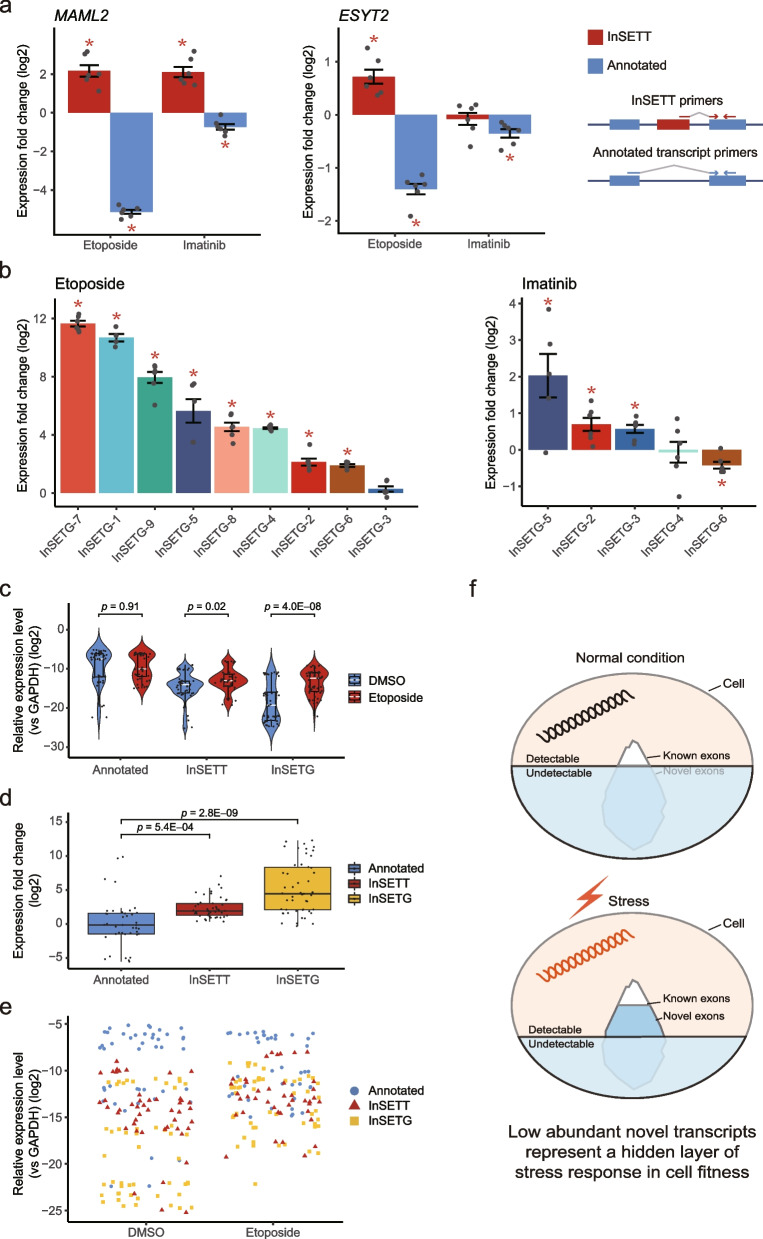
Expression analysis of novel transcripts and genes. **a** The log_2_ expression fold changes of *MAML2* and *ESYT2* novel and canonical transcripts following etoposide or imatinib treatment compared to the DMSO-treated control (left and middle). (Right) Red and blue arrows indicate the RT-qPCR primers designed for novel and canonical transcript respectively. **b** The log_2_ expression fold change of InSETGs under etoposide or imatinib treatment. **c** Comparison of the log_2_ relative expression levels of canonical transcripts, InSETTs and InSETGs following the treatments with etoposide or DMSO control. **d** The log_2_ expression fold changes of canonical transcripts, InSETTs and InSETGs under etoposide treatment compared to the DMSO-treated control. **e** The distribution of log_2_ relative expression level of canonical transcripts, InSETTs and InSETGs before and after etoposide treatment. **f** Illustration of the hypothetical model proposed in this study to explain expression level and functionality of transcripts containing novel functional exons. Under normal growth condition, the expression level of novel transcripts is below the detection limit explaining why they remain unannotated. With stress, their expression level increases significantly, presumably to improve cell fitness and survivability. Thus, these transcripts represent a hidden layer of elements required for cellular fitness in response to stress. For **a**, **b**, error bars indicate the SE of a total of 6 technical replicates corresponding to 2 biological replicates. Asterisks indicate significant differences under two-sided paired Student’s *t* test (*p* value < 0.05). Panels **c–e** are based on RT-qPCR experiments (a total of 6 technical replicates corresponding to 2 biological replicates, each dot represents a technical replicate) of all novel transcripts and genes and known genes profiled in this study. For InSETTs, only the ones with connection to known genes were included. In the boxplots, center lines indicate median; box limits indicate upper and lower quartiles; whiskers extend from the box limits no more than 1.5× interquartile range. Two-sided Welch’s *t* test was performed with the *p* values shown in the figures. Source data are provided in Additional file 2: Tables S18 and S19

Transcripts with bona fide function in response to anticancer drug treatments would be expected to be induced by these treatments. To investigate whether this is indeed the case, we tested response of spliced transcripts corresponding to 20 InSETes (9 intragenic and 10 intergenic) to either imatinib or etoposide using RT-qPCR (Fig. 7 and Additional file 2: Tables S18 and S19). In the case of the novel transcripts corresponding to the 3 internal cassette exons, relative changes of these transcripts were compared with those for the annotated isoforms as illustrated on Fig. 7a for *MAML2* and *ESYT2* genes and Additional file 1: Fig. S6a for *DNAH8*. Interestingly, we found that etoposide or imatinib treatments could induce specific inclusion of the novel exons as shown on Fig. 7a. For example, transcripts containing novel exons of *MAML2* genes increased 4.5- and 4.3-fold in response to etoposide and imatinib respectively while the annotated transcripts were downregulated by 35.0 and 1.7-fold (Fig. 7a). Thus, the fraction of the novel *MAML2* transcripts increased by 157.5 and 7.3-fold relative to the annotated ones in response to etoposide or imatinib. The transcripts containing novel exons of *ESYT2* genes increased 1.6-fold in response to etoposide while the annotated transcripts were downregulated by 2.6-fold (Fig. 7a). Thus, the fraction of the novel *ESYT2* transcripts increase by 4.2-fold relative to the annotated ones in response to etoposide. On the other hand, although the transcript corresponding to the novel exon in the *DNAH8* locus was also upregulated by etoposide, the upregulation of the annotated transcript in response to the treatment was much higher (Additional file 1: Fig. S6b). Thus, transcripts corresponding to all 3 novel internal cassette exons were upregulated, with 2 out of 3 exons in *MAML2* and *ESYT2* loci being specifically incorporated into novel transcripts in response to the drug treatment. Furthermore, 4 other tested alternative isoforms of known transcripts were induced by either etoposide or imatinib (Additional file 1: Fig. S7a-d). Among the 2 novel transcripts on the same strand as genes, only 1 was induced by the drugs (Additional file 1: Fig. S7e, f). Thus, 8/9 or 88.9% of the novel transcripts corresponding to the intragenic InSETes were upregulated by at least one of the drugs.

On the other hand, etoposide and imatinib had far more drastic upregulation effects on the transcripts corresponding to the novel genes (InSETGs) containing the intergenic InSETes (Fig. 7b). Of the 9 InSETGs tested, 8 and 3 were induced by etoposide and imatinib respectively with the corresponding fold changes of expression being in the ranges of 3.7–3213.7 and 1.5–4.1 respectively (Fig. 7b). The median fold induction for the two drugs were 36.8 (etoposide) and 1.6 (imatinib). Strikingly, transcripts corresponding to 2 novel genes were induced over 1000-fold while RNAs derive from 10 novel genes were induced over 2-fold by the etoposide treatments. Thus, even though under normal growth conditions, transcripts from 5/9 (55.6%) of the genes would not be detectable even by real-time PCR, these transcripts become readily detectable under stress caused by the anticancer drug treatments.

Overall, compared with the annotated transcripts, the novel transcripts and genes harboring IACFs or CACFs had much lower basal expression level before the treatments with drugs (Fig. 7c). However, they showed significant induction in response to drugs while the annotated transcripts did not (Fig. 7c–e). This resulted in the expression levels of the novel transcripts approaching those of the annotated transcripts (Fig. 7e). These results suggest that the low abundant novel transcripts harboring IACFs or CACFs represent a hidden layer of response to cellular stress, as illustrated in Fig. 7f.

### Transcripts harboring novel exons can be translated as bi-cistronic messages

As mentioned above, of the 19 intragenic InSETes for which both 5′ and 3′ RACE data were available, 13 corresponded to multiple novel isoforms of the corresponding genes (Figs. 4 and 5, Additional file 1: Figs. S3-S6). Interestingly, most of these transcripts contained either truncated or disrupted versions of the canonical, annotated ORFs. For example, as described above, *MAML2* and *ESYT2* loci contained novel cassette InSETes specifically included in mature transcripts in response to stress. Inclusion of both of these exons created in-frame stop codons in both mRNAs, thus truncating the corresponding ORFs and introducing stop codons before exon-exon junctions (Additional file 1: Figs.S9 and S10), making the corresponding non-canonical transcripts candidates for nonsense-mediated decay (NMD) [50]. In fact, multiple similar non-canonical transcripts have been annotated as NMD candidates in genomic databases [46], while the corresponding novel exons akin to InSETes that lead to generation of such transcripts are generally considered pseudoexons, and both such transcripts and exons are usually discarded as non-functional entities generated by aberrant splicing [51].

On the other hand, evidence of effect on cellular fitness in both of these InSETes as well as the stress-induced inclusion of the exons into mature transcripts prompted us to consider a possibility that such transcripts might indeed have some functions. Sequence analysis suggested one possible mechanism that could account for this — production of 2 separate proteins from each of these transcripts. Specifically, inclusion of the 855 bp novel cassette *MAML2* exon disrupted main ORF resulting in a transcript with two major ORFs: a shorter ORF1 starting with the original ATG of the canonical *MAML2* transcript and a longer ORF2 starting from a distal ATG codon at position 2825 encoding respectively proteins of 175 and 929 aa (Figs. 5a and Additional file 1: Fig. S9). On the other hand, the novel cassette exon of *ESYT2* corresponded to multiple (as many as 160) transcript isoforms (Fig. 5b and Additional file 1: Fig. S5), partly caused by variation in the splicing patterns of the novel exon itself. Coding potential based on different versions of the novel exons also revealed similar disrupted ORFs as in the case of *MAML2*. An example of one such case containing the 180 bp novel exon (arrow, Fig. 5b) is shown in the Additional file 1: Fig. S10. Just like in the case of *MAML2*, the insertion of the new exon created a shorter ORF1 starting with the canonical ATG and a longer ORF2 starting with distal ATG at position 916 encoding respectively proteins of 284 and 677 aa (Additional file 1: Fig. S10).

**Fig. 8:**
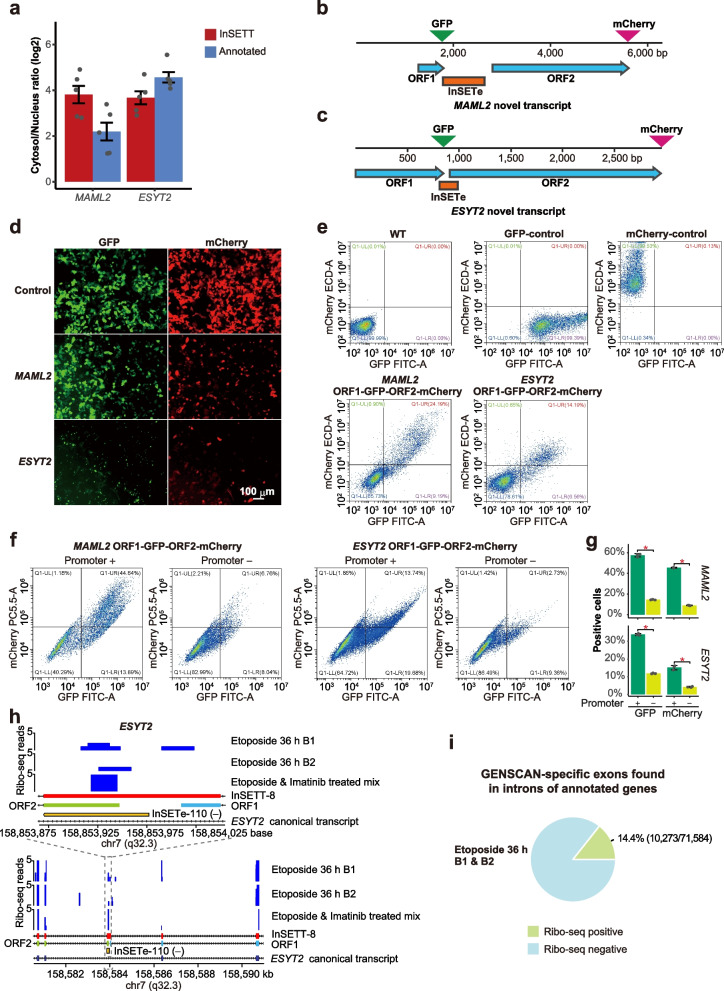
In vivo and in silico characterization of the protein-coding potential of InSETTs and InSETGs. **a** The log_2_ cytosol/nucleus ratio of the novel and annotated transcripts of *MAML2* and *ESYT2* in the presence of etoposide. Error bars indicate the SE based on a total of 6 technical corresponding to 2 biological replicates. All data are shown relative to the log_2_ cytosol/nucleus ratio of a nuclear-localized vlincRNA which is set to zero. **b**, **c** Schematic diagram of in silico predicted major ORFs in the InSETTs formed by the inclusion of the novel cassette InSETes in the *MAML2* and *ESYT2* loci chosen for these experiments (see Additional file 1: Fig. S9 and S10 for more details), and positions of the in-frame fusions of the GFP and mCherry proteins in those ORFs. Blue arrows indicate predicted ORF1 and ORF2, orange boxes indicate the novel exons. Green and magenta triangles indicate the position where GFP and mCherry proteins were inserted. **d**, **e** Fluorescence microscopy images and flow cytometry analysis of 293FT cells transfected with vectors harboring the GFP/mCherry fusions for the *MAML2* or *ESYT2* novel transcript. Scale bar, 100 mm. 293FT cells without transfection and those transfected with vectors expressing either only GFP or mCherry serve as the negative and positive controls respectively for gating. Only one representative biological replicate is shown — for the results from the other 2 biological replicates see Additional file 1: Fig. S11c. **f** Flow cytometry analysis of 293FT cells transfected with vectors harboring the GFP/mCherry fusions for the *MAML2* or *ESYT2* novel transcript with and without the CMV promoter. Only one representative biological replicate is shown — for the results from all 3 biological replicates see Additional file 1: Fig. S12. **g** Quantitation of the results of 3 biological replicates of experiments shown in the panel **f**, error bars represent SD. Asterisks show significant differences per two-sided paired Student’s *t* test (*p* value < 0.05). **h** Signals from the Ribo-seq assays for the two biological replicates of K562 cells treated with etoposide for 36 h and one replicate of pooled etoposide- and imatinib-treated cells (see “ Methods” for more details) are shown. The top portion represents zoom-in view of the ORF1 and ORF2 for the novel transcript in the *ESYT2* locus (InSETT-8) that was used for the GFP/mCherry fusions. **i** Percentage of GENSCAN-specific exons found in introns of annotated genes containing Ribo-seq signal in both biological replicates of the etoposide-treated cells. Source data are provided in Additional file 2: Table S20

While mammalian genomes do encode polycistronic RNA, they are still quite rare and represented by only 13 known examples [52]. And, even though, widespread translation of short upstream ORFs found in mammalian transcripts has been shown recently, such ORFs encode short peptides [53] (see “Discussion”) — much shorter than the predicted products of the ORF1 and ORF2 of *MAML2* and *ESYT2*. Still, to test the possibility that these transcripts could be translated, we first estimated the relative levels of both the novel and annotated transcripts of the same gene in cytosol and nucleus in the presence of etoposide. In these experiments, we used a non-coding vlincRNA exclusively localized to nucleus as a control. The cytosol/nucleus ratio of the novel isoforms of *MAML2* and *ESYT2* were similar to those of the corresponding canonical transcripts and 14.1- and 12.7-fold higher than the nuclear-localized vlincRNA (Fig. 8a and Additional file 2: Table S20). These data suggested that the novel non-canonical isoforms are exported to cytosol at the levels similar to the canonical isoforms and as such, have the potential to be translated into proteins.

To directly test whether these transcripts could represent bi-cistronic mRNAs, we created in-frame fusions of ORF1 and ORF2 in both *MAML2* and *ESYT2* with GFP and mCherry respectively, as shown in the Fig. 8b, c and Additional file 1: Fig. S11a, b. Expression of both ORF1 and ORF2 in the same cells was assayed using fluorescence microscopy and flow cytometry in 3 independent biological replicates (Fig. 8d, e and Additional file 1: Fig. S11c). Interestingly, majority of the cells expressing either GFP or mCherry expressed both proteins: in the biological replicate shown on the Fig. 8e, 24.19 and 14.19% of cells transfected with *MAML2* or *ESYT2* fusion constructs expressed both proteins. The population of the cells expressing only GFP (9.19 and 6.56% for *MAML2* and *ESYT2* respectively) was larger than that of the cells expressing only mCherry (0.9 and 0.65% for *MAML2* and *ESYT2* respectively), consistent with more efficient translation of the first ORFs (Fig. 8e). These results were consistent among all 3 biological replicates (Fig. 8d, e and Additional file 1: Fig. S11c). To confirm that both ORFs were in fact translated from the same message, and exclude a possibility of cryptic promoters present in the sequences upstream of the first ATG codons of the 2nd ORFs, we modified the plasmids expressing the novel non-canonical *MAML2* and *ESYT2* transcripts by removing the upstream CMV promoter sequence used to drive their expression. The deletion dramatically reduced translation of both 1st and 2nd ORFs, as shown by the significant reduction of GFP and mCherry positive cells for both *MAML2* and *ESYT2* (Fig. 8f, g and Additional file 1: Fig. S12). These results confirm that both ORFs of both genes are primarily translated from the same mRNAs.

To further validate the bi-cistronic nature of these novel transcripts, ribosome profiling (Ribo-seq) experiments were performed with cells treated with etoposide or imatinib as described in the “ Methods” section. Briefly, the 3 Ribo-seq samples contain two biological replicates of K562 cells treated with etoposide for 36 h, and one pooled sample with various time of either etoposide or imatinib treatment. Since the ORF1 and ORF2 of *MAML2* novel transcript completely overlapped with the ORF from the canonical transcript (Additional file 1: Fig. S9), it was not possible to specifically assign the Ribo-seq signal to either canonical or novel ORFs. On the other hand, ORF1 and ORF2 of *ESYT2* contained unique sequences (Additional file 1: Fig. S10) that could be used to differentiate them from the main canonical ORF. As shown in Fig. 8h, we could consistently observe the expected specific enrichment of the Ribo-seq signal in the annotated protein-coding exons of *ESYT2* relative to the flanking intronic sequence. We could also detect enrichment of the Ribo-seq signal precisely at the beginning of the ORF2 of the novel non-canonical transcript in all 3 samples, thus providing additional supporting evidence that the ORF2 is indeed translated.

Overall, we observed Ribo-seq signal in 10,273 out of 71,584 or 14.4% of intragenic exons predicted only by GENSCAN and not found in the current annotations in both biological replicates of the etoposide-treated K562 cells (Fig. 8i). The existence of thousands of predicted exons that could potentially be used for translation — in just one cell line and one stress condition — suggests that cellular proteome could be far more complex than anticipated. While the Ribo-seq results cannot directly answer how many of these exons are parts of bi-cistronic messages, the fact that at least 2 out of 24 such exons, for which full transcripts structures were elucidated, appear to represent bi-cistronic transcripts, suggests that bi-cistronic translation might be more common in humans than hitherto believed.

## Discussion

Here, we investigated performance of lentivirus-based insertional mutagenesis as an unbiased forward genetics tool, and applied it to investigate the landscape of functional elements in the human genome. One of the advantages of an unbiased forward genetics screen is that it does not depend on functional annotation of genomic elements while reverse genetics screens, based on for example RNAi or CRISPR/Cas9, require previous knowledge of potential functional elements to target. Insertional mutagenesis is well-suited for survey of both known and novel functional genomic elements. However, while being technically straightforward, lentivirus-based mutagenesis strategy has a number of potential problems. First, only single copy of a target element is likely to be inactivated in the genome, resulting in a partial knockdown which could present potential problems in phenotypic screens, especially in aneuploid cells typically used by the community. So far, this consideration has limited application of this strategy to very few haploid cell lines [30–33]. Second, there is an absence of validated bioinformatics pipelines to identify genomic coordinates of insertions that can cause phenotypes. Third, integration events disrupting elements critically important for survival could be lost during the generation of the insertional mutagenesis cell library and, therefore, not detected in phenotypic screens. Fourth, the most important issue that could plague this approach is that phenotypes associated with lentiviral insertions could be caused by affecting elements other than those located at the sites of the insertions, for example, by transactivating nearby genes via strong enhancer or promoter elements present in the lentiviral genome, representing an off-target effect of this method. Therefore, even though the site of viral integration could be unambiguously mapped in a genome, the association between a phenotype and a specific genomic location may be ambiguous.

To our knowledge, this work represents the first study in which most of these concerns were thoroughly investigated and addressed as follows. First, we showed that even in an aneuploid cell line, the lentiviral integrations can have phenotypic effects as evidenced by significant loss of integrations in the exons of essential protein-coding genes and lncRNAs found previously in two independent CRISPR/Cas9 screens. Furthermore, cross-confirmation of the results from these screens for both mRNAs and lncRNAs is an important corollary of this work. Especially in the case of the latter, since biological functions of lncRNAs are not yet universally accepted [21]. Supporting the results from the previous CRISPR/Cas9 screen using independent insertional mutagenesis approach provides additional validation for the functionality of these transcripts.

Second, we have tried two independent analytical approach to detect phenotypic insertions based either on individual insertion events or on regions of nearby insertions that show similar phenotypic effects. We additionally validated the performance of the latter approach by using simulated datasets. Overall, we found significant agreement between the two approaches both in terms of the positions of phenotypic insertions detected by the two methods and in terms of the overall conclusions. However, we also found that the region-based analytical approach was far more sensitive. Therefore, we propose that such approach, either based on SICER or some other algorithm, should be used in similar lentiviral screens.

Third, as expected, we have indeed encountered a low power of the method in detection of critically essential functional elements as evidenced by three observations. One, we have found depletion of insertions in genes found to be essential in the CRISPR/Cas9 screens even after very short (2 days) periods of growth of the libraries. Therefore, cells with insertions in such genes were lost even before phenotypic screens, consistent with the results from previous retrovirus-mediated insertional mutagenesis screens in a haploid cell line [33]. Two, the essential genes had higher expression levels in the corresponding cell type compared to the non-essential ones. However, the genes with insertions in their exons had significantly lower expression levels than the genes without such insertions. Three, we could not detect consistent enrichment of phenotypic insertions in exons of annotated genes in all of the 4 phenotypic assay systems. Therefore, this method would most likely identify non-essential, but still biologically relevant genomic elements.

Fourth, forward genetics screens performed so far had a tendency to be focused on the annotated transcripts of known genes [30–33]. Here, we extended the scope of the analysis to the unannotated portions of the human genome, both intra- and intergenic. Strikingly, we found that most phenotypic insertions were not associated with exons of known genes, with a large fraction of them located in the intergenic space. One possible explanation of these results is that such insertions function by affecting expression of known genes, either positively by transactivating their expression or negatively by interfering with splicing or transcription. However, in this work, we provide 3 lines of arguments that suggest that this is not the case, or at least that these effects do not contribute significantly to the overall conclusions. One, our analysis of insertion distribution around essential genes suggests that transactivation, at least in our lentiviral system, happens at relatively small distances, ±10 kb, around TSSs of known genes. Two, we have not detected obvious negative phenotypic signature from insertions mapping to introns of essential genes that would be expected if these insertions affected proper processing or transcription of these genes. Three, if most of phenotypic insertions act by affecting expression of annotated genes, we would have expected to observe enrichment of these insertions in introns and/or around TSSs of known genes compared to insertions that have no phenotypes. However, this trend has not been observed. Still, the potential indirect effects of viral insertions should be carefully considered and characterized in the future similar insertional mutagenesis screens.

Taken together, our results argue that the phenotypic insertions located outside of exons or ±10-kb windows around TSSs of known genes affect multiple, yet unannotated functional elements in the human genome. This conclusion was further supported by the sequence conservation analysis: if phenotypic insertions were tagging unannotated functional elements, the sequences at the sites of those insertions would be expected to be significantly more conserved than those at the sites of insertions with no phenotypes, and this was indeed observed. While it is quite likely that the method identified multiple types of functional elements, our findings suggest that at least some of these elements are represented by novel exons. Indeed, we could observe consistent enrichment of phenotypic insertions in unannotated exons predicted only by GENSCAN and located inside known genes. Disruption of these novel exons is consistent with the clear phenotypic signature of insertions in exons of essential genes, either of protein-coding genes or lncRNAs, that we could detect in this work, and is also consistent with the expected disruptive effect of insertion of the lentiviral genome in these genomic elements. As we have shown using RACE analysis, many of such predicted exons do represent bona fide exons of the corresponding genes or exons of transcripts sharing the same genomic region with the genes. In addition, in some assays, phenotypic insertions are also enriched in the unannotated GENSCAN-predicted exons located in the intergenic space that as we have also shown can correspond to novel genes encoding spliced transcripts. These results are consistent with the observation that over 90% of all hits from genome-wide association studies (GWAS) map outside of exons and about half map outside of genes [54, 55]. In fact, intergenic regions in general, including those containing GWAS hits, have been shown to express many unannotated transcripts including lncRNAs [56–58], and it has been hypothesized that disease- or trait-associated sequence variants uncovered in GWAS studies might function by affecting novel transcripts and genes [59–61].

Overall, these results point — consistent with the pervasive presence of the “RNA dark matter” in the human genome [2–4] — to the existence of a potentially large pool of functional exons corresponding to transcripts and genes that are yet to be annotated. For example, the GENSCAN program can correctly predict 86.2% of base pairs that constitute protein-coding portions of annotated exons in the current GENCODE database. However, these sequences correspond to only 28.2% of all base pairs predicted by GENSCAN to represent human exons. Therefore, the majority of exonic sequence predicted by GENSCAN program, corresponding to 345,175 exons not found in the GENCODE genome annotation database, are considered to be false positives. On the other hand, our results suggest many of these GENSCAN-specific exons do exist and could be functional. Based on the results presented in this work, we estimate that of those, at least ~15K GENSCAN-specific exons might represent novel functional genes encoding spliced transcripts (278,590 total intergenic GENSCAN-specific exons × 9.5% (397/4,165) intergenic exons harboring either IACFs or CACFs × 58.3% (14/24, Fig. 4g) spliced transcripts) and ~1.4K represent novel functional internal cassette exons of protein-coding genes (71,584 total intragenic GENSCAN-specific exons × 9.7% (295/3035) intragenic exons harboring either IACF or CACFs × 20.8% (5/24, Fig. 4g) internal exons). This estimate does not include intergenic exons that represent unspliced transcripts as well as exons representing novel initiation and termination exons of known genes, and exons of novel transcripts on the same strand as known genes as exemplified in this work (Fig. 4g). Furthermore, since only two cell types were used in this study and the phenotypes were screened in cultured cells, many elements that are conditionally functional in different cell types, conditions, and developmental stages might be missed. This is especially true for lncRNAs that are known to be highly cell-type specific [41, 62, 63]. Furthermore, not all novel exons can be predicted by GENSCAN. Therefore, the true number of novel biologically relevant exons could be much higher. These conclusions suggest that future forward and/or reverse genetics screens should also target in silico predicted exons to determine what fraction of them is essential, and screens under different biological conditions and contexts will be informative.

In this respect, it is worth mentioning that lncRNA transcripts and genes are widespread [64] and contribute to the majority of mammalian non-ribosomal nuclear transcriptome by mass [65], and many of these transcripts and genes remain to be discovered and annotated [17, 64]. Furthermore, many of the ones that have been discovered have not yet been annotated in GENCODE [66–69]. For example, the class of enhancer RNAs (eRNAs), which are transcribed from enhancer sequences, is emerging as an important component of non-coding transcriptome and these transcripts may participate in regulation of enhancer activity [70]. However, as recently shown, tens of thousands of eRNAs are not annotated in GENCODE [71]. Therefore, the lncRNA annotations used here represent only a minor portion of all lncRNAs. Therefore, the IACFs and CACFs outside of the annotated elements could very likely affect unannotated lncRNAs, including eRNAs.

One of the main conclusions from this work is that it is likely that many noncanonical transcripts derived from annotated protein-coding loci that were previously discarded as aberrant splicing products can in fact be translated as bi-cistronic messages and are biologically relevant. These results are consistent with a number of recent reports that have shown that mammalian proteome is much more complex than previously anticipated [53, 72]. First, mass spectrometry analysis has shown that multiple novel proteins and peptides could be produced from non-coding genomic space [72]. Second, Ribo-seq assays revealed widespread protein-coding capacity of transcripts previously considered as non-coding, and moreover, presence of complex arrangements of ORFs in protein-coding mRNAs [53]. For example, these experiments have shown common presence of bi-cistronic mRNAs containing short upstream, and also, in fewer cases, downstream ORFs in addition to the major ORFs [53]. In fact, recently, the same group has shown using CRISRP/Cas9 screen that upstream (u)ORFs are translated together with the canonical ORFs and encode functional peptides [73]. However, most of the uORF reported previously encode short peptides or microproteins of <100 aa [74]. As mentioned above, polycistronic translation is still considered to be very rare in eukaryotes with only 13 polycistronic mRNAs found in mammalian genomes [52]. Overall, these results suggest that additional studies into discovery and functional annotation of novel isoforms of annotated human genes are well-warranted. Also, they suggest it might be worth to reconsider some of the general principles of annotation of transcripts in the human genome, since such bi-cistronic transcripts would be typically categorized as non-functional substrates of NMD [46], and the exons whose inclusion interrupts ORFs, similar to multiple InSETes identified here, are currently often considered as non-functional pseudoexons [51]. It is important to emphasize that while in this work we found unusual alternative novel transcripts and bi-cistronic translation events, they cannot be simply attributed to the cancerous nature of the cells used in this study. In fact, complex patterns of multiple types of novel and non-canonical transcripts have been shown to be widely present in normal mammalian cells and tissues [49, 58, 64, 75, 76] (reviewed in [77]). Furthermore, several previous reports support the existence of non-canonical translation events, including bi-cistronic or polycistronic transcripts, in normal cells [53, 73, 74]. The relevance of the novel transcripts detected in this study in normal cells is further supported by the detection of expression of most of them in normal human tissues and primary cells.

Interestingly, specific inclusion of the novel exons that generates these isoforms happens in response to treatments with anticancer drugs etoposide (*MAML2* and *ESYT2*) or imatinib (*MAML2*). Alternative splicing has been shown to play an important part in anticancer drug resistance, leading to the emergence of new therapeutic strategies that combine treatments with splicing modulators and therapeutic agents [78, 79]. However, the number of known alternative splicing events caused by anticancer drugs is still limited [79]. Considering that thousands of un-discovered functional cassette and other types of exons exist and that the majority of novel intragenic exons tested here are preferentially included into the mature transcripts in response to drugs, the functional alternative splicing caused by anticancer drug treatments can actually be quite prevalent. Combined with the bi-cistronic feature of the drug-induced novel non-canonical isoforms, this calls for further exploration of alternative splicing and new proteome generated by it as an important new dimension in cancer therapy.

However, as shown above, the depth of long-read sequencing techniques is still not sufficient to comprehensively discover low abundant transcripts containing novel exons. For example, only 1 out of 131 InSETes was detected by PacBio RNA-seq compared to 90 detected using the RACE enrichment approach. Similar conclusions were also reached by other groups that employed targeted transcript enrichment techniques followed by NGS [64, 80]. However, the low abundance of such exons in a bulk RNA sequencing does not necessarily mean that these transcripts are non-functional since, as shown by single-cell transcriptome profiling studies, transcripts with low abundance in bulk samples could have much higher expression levels in individual cells [81, 82], even in the case of supposedly homogenous cultured K562 cell line [82]. To overcome the abundance issue, RACE or other targeted transcript enrichment techniques could be applied to identify transcripts containing novel exons of interest, for example predicted by GENSCAN or any other program and shown to be functional in a forward- or reverse genetics screen, as illustrated in this work for InSETes.

## Conclusions

All in all, our results suggest that the functional landscape of the human genome still remains to a large extent unexplored with multiple physiologically relevant exons, transcripts, and genes remain to be discovered and characterized. In particular, we show that thousands of human exons predicted purely in silico could likely correspond to bona fide exons of spliced and functional transcripts. In this respect, we believe that one of the most important outcomes of this work is the realization that in silico exon- or gene-prediction tools are still very much relevant for genome annotation efforts. The development of such approaches, while very popular in the 1990s, has become much less popular in the age of RNA-seq. However, our results based on the GENSCAN exons show that such in silico prediction tools, potentially in combination with a more powerful modern deep-leaning methods, could represent powerful approaches to identify novel exons and should be pursued alongside the wet lab approaches in the genome annotation efforts for both protein-coding and non-coding transcripts. The combination of many exons of annotated lncRNAs with the powerful artificial intelligence (AI) solutions available today make this a very tenable endeavor even for non-coding exons. As such, a combination of insertional mutagenesis or other high-throughput reverse genetics approach, exon predictions, and RACE can be a very powerful approach for genome annotation.

Furthermore, our results raise a possibility that multiple non-canonical transcripts that so far have been discarded could in fact have function, for example, as bi-cistronic mRNAs. Altogether, this study strongly suggests that future efforts to systematically annotate transcripts containing in silico exons using RACE or other targeted transcript enrichment techniques and reverse genetics approaches that target such exons are well-warranted in order to fully understand the complexities of both protein-coding and non-coding transcripts harbored by the genome.

## Methods

### Biological resources

Human liver hepatocellular carcinoma cell line HepG2 and human chronic myeloid leukemia cell line K562 were obtained from Cell Bank of Chinese Academy of Sciences and maintained in RPMI 1640 medium (Gibco) supplemented with 10% fetal bovine serum (ExCell Bio, Uruguay) and 1% penicillin-streptomycin (Gibco) at 37℃ in 5% CO_2_.

### Insertional mutagenesis

To generate insertional mutagenesis cell libraries HepG2.LTR and K562.LTR used in the screens for essential genomic elements in cell growth and survival, HepG2 and K562 cells were transfected with lentiviral particles generated using the lentivirus vector pLV-hef1a-mNeongreen-P2A-Puro-WPRE-CMV-MCS-3Xflag in a 293FT packaging cell line with an MOI of 20. Three independent transfections were performed for each cell line. Forty eight hours after transfection, the 3 replicates were combined to perform flow cytometry (BD FACSAria III, BD Biosciences, USA) sorting with mNeongreen as the selection marker to obtain more than 6 × 10^6^ cells with lentivirus insertions per cell line. The cells were then divided into 6 equal parts. Three samples from each cell line were subjected to DNA isolation with TIANamp Genomic DNA Kit (Tiangen) and served as the controls. The other 3 samples were further cultured for 1 month and over 1 × 10^6^ cells were harvested and used for DNA isolation.

To generate insertional mutagenesis cell library K562.LTR2 for the anticancer drug survival challenge screen, K562 cells were consecutively transfected at 15–20 MOI with lentiviral particles generated with plasmids pLV-TRE-NLS-hcas9-NLS-T2A-EGFP and pLV-CMV-mcherry-P2A-rtTA in a 293FT packaging cell line. K562 cells transfected with the first vector were selected by flow cytometry (BD FACSAria III, BD Biosciences, US) using GFP as the selection marker, expanded and transfected with the second vector and selected by flow cytometry using mCherry. These cells were then further expanded for 3 months. The generation of insertional mutagenesis cell libraries HepG2.LTR, K562. LTR, and K562.LTR2 was outsourced to SyngenTech Corporation (Beijing).

Two million K562.LTR2 cells were seeded into each well of a 6-well plate with 2 ml medium containing either 0.5 μM imatinib (AbMole BioScience, US) or 40 μM etoposide (AbMole BioScience, US). Cells were treated for 48 and 24 h with imatinib and etoposide respectively, then washed twice with 1 ml RPMI 1640 to remove the drugs and resuspended in 2 ml fresh medium for recovery. During the recovery, cells were passaged daily with the maximum density of 1 × 10^6^ cells/ml, and 45 μl of cells was collected and mixed with 45 μl of medium and 10 μl of 0.4% trypan blue staining solution (Solarbio) to evaluate the fraction of the live cells. The next round of drug treatment was performed when most cells recovered the normal shape or the doubling rate of the untreated cells. According to the recovery kinetics of imatinib and etoposide, 5 and 1 rounds of drug treatment and recovery were performed for each drug respectively. Three independent biological replicates were performed for each treatment. DNA was harvested in cells before and at the end of the treatment using TIANamp Genomic DNA kit (Tiangen).

### Detection of lentivirus insertion sites

Lentivirus insertion sites in the genome were detected as described in Additional file 1: Fig. S1a. Briefly, 1 μg of genomic DNA was used as the template for a linear PCR in a 50 μl reaction system containing 1× Taq Buffer, 2.5 U Taq DNA polymerase (Tiangen), 4 μl of 2.5 mM dNTP mix (Takara), and 0.3 μM biotinylated primer 3-LTR_prime (5′-biotin-GCTCAACTGGTACTAGCTTGTAGCACCATCC-3′), which anneals to the 3′ LTR region of the lentivirus vector. Fifty cycles of linear PCR were performed with 2.5 U Taq DNA polymerase (Tiangen) added at the beginning and immediately after 25 cycles of the reaction. PCR conditions were as follows: 94℃ for 5 min; 50 cycles of 94℃ for 30 s, 55℃ for 30 s and 72℃ for 30 s; 72℃ for 5 min. After the linear amplification, the PCR products were mixed with 10 μl of BeaverBeads™ Streptavidin (Beaver) and incubated for 2 h at room temperature with shaking (400 rpm) in a metal bath. Beads were then collected with a magnetic stand and washed with 300 μl Binding/Wash buffer (10 mM Tris-HCl (pH 7.5), 1 M NaCl, 1 mM EDTA, 0.1% Tween-20) for 6 times, and with 65°C H_2_O for twice. For the second strand synthesis, beads were resuspended in a 24 μl reaction volume containing 1× Klenow Fragment Buffer (NEB), 2 μl of 2.5 mM dNTP mix (Takara), 6.25 μM P5_N6 primer (5′-CTACACGACGCTCTTCCGATCTNNNNNN-3′) and pre-incubated at 15℃ for 20 min. Then, 2 U of Klenow polymerase (NEB) were added and the mixtures were incubated at the following conditions: slow ramp at 0.5℃ per minute from 15 to 25℃ followed by 30 min incubation; then slow ramp at 0.5℃ per minute to 37℃ followed by 1 h incubation. Beads were captured with a magnetic stand and washed gently with 300 μl of 4℃ water. To construct the library for NGS, nested PCR was performed. First, beads were resuspended in a 25 μl PCR reaction volume containing 1× Taq Buffer, 1.25 U Taq DNA polymerase (Tiangen), 2 μl of 2.5 mM dNTP mix (Takara), 0.5 μM of primer P5 (5′-CTACACGACGCTCTTCCGATCT-3′), and 0.5 μM of primer 3-LTR_Nest (5′-CCTGGTGTGTAGTTCTGCCAATCAG-3′). PCR conditions were as follows: 94℃ for 3 min; 20 cycles of 94℃ for 30 s, 55℃ for 30 s and 72℃ for 30 s; 72℃ for 7 min. Second, 5 μl of the PCR products from the previous step were used for the next round of PCR in 50 μl volume containing 1× Taq Buffer, 2.5 U Taq DNA polymerase (Tiangen), 4 μl of 2.5mM dNTP mix (Takara), 0.4 μM of primer Illumina_P5 (5′-AATGATACGGCGACCACCGAGAtctACACTCTTTCCCTACACGACGCTCTTCCGATCT-3′) and 0.4 μM of primer Illumina_P7-3LTR (5′- CAAGCAGAAGACGGCATACGAGATCGTGATGTGACTGGAGTTCAGACGTGTGCTCTTCCGATCTGCCTTGTGTGTGGTAGATCCACAG-3′). PCR conditions were as follows: 94℃ for 3 min; 15 cycles of 94℃ for 30 s, 55℃ for 30 s and 72℃ for 30 s; 72℃ for 7 min. PCR products were purified with 1.2× volumes of VAHTS DNA Clean Beads (Vazyme) to a final volume of 21 μl. The concentration of PCR products was measured by Qubit 3.0 fluorometer using Equalbit dsDNA HS Assay Kit (Vazyme). To achieve a better coverage, five libraries were prepared in parallel from 1 mg of genomic DNA and pooled together. NGS was performed on Illumina platforms HiSeq X Ten or NovaSeq 6000 using paired-end 150 bp (PE150) strategy and outsourced to Novogene Corporation (Beijing) on 30-GB (giga-base) scale for each replicate of HepG2.LTR and K562.LTR libraries and 33-GB scale for each replicate of K562.LTR2 libraries.

Only paired-end raw reads with the read 2 starting with LTR tag “GCCTTGTGTGTGGTAGATCCACAGATCAAGGATATCTTGTCTTCGTTGGGAGTGAATTAGCCCTTCCA” and each base of each read having Phred quality score > 20 were selected. Such reads were aligned to the GRCh37/hg19 using BWA-MEM (v0.7.12) with default settings. Only read-pairs where both read 1 and 2 uniquely mapped to the genome with appropriate configuration and spacing were kept. The insertion position was defined as the first base upstream of the LTR tag in the read 2.

### RNA-seq analysis

We used previously published RNA-seq data from normally grown K562 cells generated by our group [83, 84]. In that study, RNA was isolated from K562 cells and RNA-seq was performed on Illumina platform (HiSeq X Ten) by Novogene Corporation (Beijing) using rRNA-depletion protocol and PE150 strategy on 10-GB scale. Two biological replicates were performed. To calculate the TPM of genes, the raw reads were trimmed with fastq_quality_trimmer of the FASTX-Toolkit (v0.0.13) software to obtain paired-end reads with a Phred quality score ≥ 20 for each base. The TPM was calculated based on the UCSC Genes track from the GRCh37/hg19 assembly of the UCSC Genome Browser [44] by the RSEM (v1.2.28) software with parameters “--bowtie2 --paired-end --strand-specific --no-bam-output.” The statistical significance difference between TPM of CS > 0 and CS < 0 genes were calculated using two-sided paired Student’s *t* test.

### Identification and analysis of IACFs and CACFs

The IACFs and CACFs were identified as illustrated in Additional file 1: Fig. S1b. The read counts in each unique insertion site were normalized to the total number of uniquely aligned reads in each sample. For the identification of IACFs, the normalized read counts were converted to log_10_ values. The log_10_ values of insertion sites with zero reads were set as −5. Two-sided paired Student’s *t* test was performed on the log_10_ values. The *p* values were adjusted for multiple comparisons with the Benjamini-Hochberg method in *R* environment and IACFs were selected with an FDR threshold of 25%. In the statistical analysis, a 3 by 3 comparison was performed between the 3 biological replicates of control and treated samples to obtain significantly enriched or depleted positions. To obtain CACFs, the significantly enriched or depleted insertion clusters between control and treated samples were identified using SICER [40] (v1.1) with a FDR threshold of 1%, and the cluster regions shared by at least two biological replicates were denoted as CACFs. In HepG2.LTR and K562.LTR cell lines, 1-month-cultured samples were compared with control samples collected at 48 h after transfection; in K562.LTR2 cell line, imatinib or etoposide-treated samples were compared with control samples prior to the drug challenge.

The overlaps between insertion sites or clusters and the different genomic elements were calculated using the “intersect” function of the BEDTools suite (v2) [85]. For the analyses shown in the Figs. 3 and 4, the annotations, unless indicated otherwise, were downloaded from the following tracks and databases of the GRCh37/hg19 assembly of the UCSC Genome Browser [86]. The known genes were represented by the “UCSC Genes” database [44]. For each gene, only the longest annotated transcript (based on the total length of the exon) was chosen for the subsequent analysis. Annotations of promoters, enhancers, and insulators for HepG2 and K562 cells lines were obtained from the “Chromatin State Segmentation by HMM from ENCODE/Broad” track [42, 43]. Annotations of lncRNAs were obtained from the “GENCODE Genes” track [45, 46]. Coordinates of vlincRNAs were based on St Laurent et al. [41]. Annotations of GENSCAN [34] exons were obtained from the “Genscan Gene Predictions” track.

### Treatment of K562 cells with anticancer drugs

Detection of full-length transcript isoforms corresponding to novel GENSCAN exons or connections between them and neighboring annotated exons using 5′ and 3′ RACE, detection of exon-exon connections using RT-PCR, or RNA-seq using PacBio long-read sequencing was performed on the same RNA pool of drug-treated K562 cells prepared as follows. K562 cells (5 × 10^5^ cells/ml) were grown in RPMI 1640 (Thermo Fisher Scientific, US) supplemented with 10% fetal bovine serum (ExCell Bio, Uruguay) in a 6-well plate for 16 h and treated with either 80 μM etoposide or 1 μM imatinib for 0, 6, 24, or 36 h. After the treatment, RNA was isolated with TRNzol Universal (Tiangen) and E.Z.N.A. Total RNA Kit I (Omega). The polyA+ fraction was isolated from each total RNA by Library Preparation VAHTS™ mRNA Capture Beads (Vazyme). An equal amount of polyA+ RNA from each sample was mixed and used for cDNA synthesis for RACE, PacBio RNA-seq, or RT-PCR. To detect the relative levels of canonical and novel non-canonical *MAML2* and *ESYT2* transcripts in cytosol and nucleus, K562 cells were treated with 80 μM etoposide for 24 h prior to subcellular fractionation.

### Sequence conservation analysis

The phastCons scores were obtained from the Vertebrate Multiz Alignment & Conservation (100 species) track from the GRCh37/hg19 assembly of the UCSC Genome Browser [87, 88]. The conservation scores of insertion sites were extracted using the “intersect” function of the BEDTools suite [85] (v2).

### Identification of connections between intragenic InSETes and neighboring annotated exons using RT-PCR

One hundred nanogram of the pooled PolyA+ RNA (described above) was used for cDNA synthesis with PrimeScriptTM II 1st Strand cDNA Synthesis Kit (Takara) with pools of gene-specific primers. For RT-PCR, two to three rounds of nested PCR were performed. The second or third round of amplification was conducted with 2 μl products from the previous round of PCR. RT-PCR products were subjected to agarose gel analysis with the relative concentrations quantified by the Tanon 3500R Gel Imaging System (Tanon). For downstream Nanopore Technologies sequencing analysis, equal amounts of RT-PCR products from each reaction were pooled and purified with 2× volumes of VAHTS DNA Clean Beads (Vazyme). The list of RT-PCR primers can be found in Additional file 2: Table S21.

### Identification of full-length transcripts representing novel GENSCAN exons using 5′ and 3′ RACE

For the 5′-RACE, first-strand cDNA synthesis was performed using the PrimeScript™ II 1st Strand cDNA Synthesis kit (Takara) following the manufacturer’s instructions with some modifications. Briefly, 85 ng of pooled polyA+ RNA from above was used as the template. Up to ten exon-specific primers (GS) were pooled in each reaction to a final concentration of 0.5 μM for each primer. The reactions were incubated at 50℃ for 60 min, followed by enzyme inactivation at 70℃ for 15 min. The products were purified with 1.5× volumes of VAHTS DNA Clean Beads (Vazyme) to a final volume of 15 μl. For polyC tailing with terminal transferase (TdT) (NEB), the purified first-strand cDNA was mixed with 2 μl of 10× TdT buffer and 2 μl of 2.5 mM CoCl_2_, and denatured at 95℃ for 5 min followed by rapid snap-cooling on ice. The reaction was then mixed with 5 U TdT (NEB) and 2 μl of 10 mM dCTP (Takara) in a total volume of 22 μl and incubated at 37℃ for 30 min, followed by enzyme inactivation at 70℃ for 10 min. The tailing reactions were purified with 1.5× volume of VAHTS DNA Clean Beads (Vazyme) to a final volume of 15 μl. The 5′-RACE PCR was performed with all of the purified products of the tailing reaction in a 25 μl reaction system containing 1× PrimeSTAR GXL Buffer, 1.25 U PrimeSTAR GXL DNA polymerase (Takara), 2 μl of 2.5 mM dNTP mixture (Takara), 0.4 μM N10G10HN primer (5′-AGTTGCGGATGGGGGGGGGGHN-3′), and 10 pooled exon-specific nested primers (GSN) with a final concentration of 0.4 μM for each primer. The 5′-RACE PCR conditions were as follows: 30 cycles of 98℃ for 10 s, 55℃ for 30 s and 68℃ for 5 min; 68℃ for 7 min.

For the 3′-RACE, first-strand cDNA synthesis was performed using the PrimeScript™ II 1st Strand cDNA Synthesis kit (Takara) with 350 ng of the pooled polyA+ RNA and N20T12VN primer (5′-GCAATCATCGAGTTGCGGATTTTTTTTTTTTTVN-3′) at final concentration of 0.5 μM. The reactions were slowly ramped at 2℃ per minute from 37 to 50℃ and further incubated at 50℃ for 60 min. The enzyme was inactivated at 70℃ for 15 min. Two rounds of nested 3′-RACE PCR were then carried out. The first round of amplification with 2.8 μl of unpurified products of the cDNA synthesis reaction as the template was conducted in a 25 μl reaction system containing 1× PrimeSTAR GXL Buffer, 1.25 U PrimeSTAR GXL DNA polymerase (Takara), 2 μl of 2.5 mM dNTP mixture (Takara), 0.4 μM N20T12VN primer, and 10 pooled exon-specific primers (rcGSN) with a final concentration of 0.4 μM for each primer. The reactions conditions were as follows: 15 cycles of 98℃ for 10 s, 55℃ for 30 s, and 68℃ for 5 min; 68℃ for 10 min. The second round of amplification was done with 1 μl of first round PCR products as the template in a 25 μl reaction system containing 1× PrimeSTAR GXL Buffer, 1.56 U PrimeSTAR GXL DNA polymerase (Takara), 2 μl of 2.5 mM dNTP mixture (Takara), 0.4 μM N20T12VN primer, and 10 pooled exon-specific nested primers (rcGS) with a final concentration of 0.4 μM for each primer. The amplification conditions were the same as for the first round of PCR except for 30 cycles of reactions were performed. The sequence of 3′-RACE primers rcGSN and rcGS are reverse compliment to that of the 5′-RACE primers GSN and GS respectively. The list of 5′-RACE and 3′-RACE primers can be found in Additional file 2: Table S22.

The 5′-RACE and 3′-RACE products were subjected to agarose gel analysis with the relative concentrations measured by the Tanon 3500R Gel Imaging System (Tanon). Equal amounts of 5′-RACE or 3′-RACE products from each pooled reaction were mixed and purified with 2× volumes of VAHTS DNA Clean Beads (Vazyme) respectively. The concentrations were measured by Qubit 3.0 fluorometer using Equalbit dsDNA HS Assay Kit (Vazyme).

### Nanopore sequencing

Products of RT-PCR, 5′-RACE, and 3′-RACE were sequenced using Oxford Nanopore Technologies platform by Baocheng Corporation (Hangzhou). Sequencing library was prepared by Ligation Sequencing Kit (SQK-LSK109) (Oxford Nanopore Technologies Inc., Oxford, UK) by Baocheng Corporation (Hangzhou). The library was sequenced on a FLO-MIN106 R9.4.1 flow cell on GridION MK1 (Oxford Nanopore Technologies Inc., Oxford, UK). Five GB of raw data were obtained after 18 h of sequencing. The sequenced reads were base-called in real time using MinKNOW (v20.10.6) and integrated with Guppy (v4.2.3).

For the analysis, reads were aligned to the GRCh37/hg19 using Minimap2 (v2.17-r941) in spliced alignment mode with the command “minimap2 -ax splice --secondary=no”, and the option “--secondary=no” was used to suppress secondary alignments. Supplementary and low-quality alignments were filtered out using SAMTools (v1.10) with the command “samtools view -F0x900 -q 60”.

### PacBio long-read sequencing

PacBio long-read sequencing was outsourced to Novogene Corporation (Beijing). Briefly, to prepare for the sequencing library, polyA+ RNA fractions were isolated from wild-type K562 cells not transfected with lentiviruses and treated separately by imatinib or etoposide for varying amounts of times and then mixed prior to the PacBio library construction as described above and reverse-transcribe to cDNA with SMARTer PCR cDNA Synthesis Kit (Clontech Laboratories). The cDNA was PCR amplified and size selected with the BluePippin Size-Selection System (Sage Science). The sequencing library was prepared with SMARTbell Express Template Prep Kit 2.0 (Pacific Biosciences). The sequencing was performed with Sequel II Sequencing Kit 2.0 on PacBio Sequel platform on 30-GB scale by Novogene Corporation (Beijing).

### Overlap analysis of InSET TSSs with CAGE tags

The TSSs of novel transcript were extracted from the 5′ RACE data for InSETes for which the 5′ RACE information was available. The coordinates of TSSs were extended by ±10 bp and either analyzed directly (Additional file 2: Table S14) or merged to obtain TSS clusters for simulation analysis (Fig. 4h and Additional file 2: Tables S15-17). For the downstream analyses, InSET TSSs overlapping annotated mRNA TSSs in ±10 bp window as well as TSS clusters overlapping annotated mRNA TSSs were filtered out. The remaining TSS clusters were mapped to exons, introns, antisense intragenic regions, and intergenic regions with a hierarchical strategy to determine the composition of the TSSs (Additional file 2: Table S15). For the simulation analysis, the “shuffle” function of the BEDTools suite (v2) [85] was used to get the simulated TSS clusters based on the distribution of TSS clusters on the abovementioned four regions in the real data. The overlap analyses between the individual TSSs or TSS clusters with CAGE tags in human tissues or primary cells were performed using the “intersect” function of the BEDTools suite (v2) [85].

For each real and simulated TSS cluster, the number of tissue or primary cell samples with overlapping CAGE tags, and the sum of normalized CAGE abundance in all of these samples were determined. The overlaps between TSSs or TSS clusters and CAGE tags were always performed in a strand-specific fashion. The normalized CAGE abundance was calculated as:

$$Normalized\;CAGE\;abundance=\frac{C\times10^7}T$$

where *C* represents the CAGE depth on each CAGE TSS, and *T* denotes the total number of CAGE tags in each sample. The statistical significance between CAGE signal of individual TSS clusters in the real and simulated data shown in Fig. 4h was calculated using Wilcoxon rank-sum test.

### RT-qPCR

For the expression analysis of novel transcripts in response to anticancer drugs, K562 cells were treated with an anticancer drug or DMSO as the control for 36 h prior to RNA isolation. For the subcellular localization analysis of the transcripts, RNA was isolated from the cytoplasmic or nuclear fractions of the K562 cells, and cDNA was synthesized using PrimeScript™ II 1st Strand cDNA Synthesis Kit (Takara). RT-qPCR was performed with PowerUp SYBR Green Master Mix (Life Technologies) on a Mx3005P cycler (Agilent Technologies) using two biological replicates each done with three technical repeats. The Ct value of an RT-qPCR replicate with no Ct value was set to 40 since 40 cycles of amplifications were performed. If one technical replicate was significantly different (>2 Ct) from the other two, it was excluded from the analysis. Two-sided paired Student’s *t* test with a 6 by 6 comparison was performed on the log_2_ values. The Hedge’s *g* effect size was calculated as:

$$Hedge's\;g=Cohen's\;d\ast\left(1-\frac3{4\left(n_1+n_2\right)-9}\right)$$

$$Cohen's\;d=\frac{\overline{X_2}-\overline{X_1}}{\sqrt{\frac{\left(n_1-1\right){SD}_1^2+(n_2-1){SD}_2^2}{n_1+n_2-2}}}$$

where *n*_*1*_ and *n*_*2*,_ $\overline{{X}_{1}}$ and $\overline{{X}_{2}}$, *SD*_*1*_ and *SD*_*2*_ are respectively the sample sizes, means, and standard deviations of the two groups. All Hedge’s *g* effect sizes in this study were calculated based on this formula. The list of RT-qPCR primers can be found in Additional file 2: Tables S23 and S24.

### Analysis of the protein-coding potential of new isoforms of known genes in vivo

ORF1 and ORF2 found by in silico translation of *MAML2* and *ESYT2* transcripts harboring the novel exons are shown in Additional file 1: Figs. S9 and S10. Sequences encoding EGFP and mCherry were inserted in-frame after the predicted ORF1 and ORF2 respectively (Fig. 8b, c and Additional file 1: Fig. S11a, b) and cloned into an expression vector driven by the CMV promoter. The resulting fusion constructs would express ORF1-EGFP and ORF2-mCherry from the same mRNA for either *ESYT2* or *MAML2* transcript containing the novel exon. The *MAML2* and *ESYT2* vectors containing CMV promoter were constructed by SyngenTech Corporation (Beijing) and GENEWIZ Corporation (Suzhou) respectively. The *MAML2* and *ESYT2* vectors without the CMV promoters were constructed by GENEWIZ Corporation (Suzhou). The fusion constructs were delivered into 293FT cells using Lipofectamine 3000 reagent (ThermoFisher Scientific, US). 293FT cells were also separately transfected with vectors overexpressing either EGFP or mCherry only (without any additional protein sequence) as the positive controls and 293FT cells without transfection served as the negative control. Cells were observed under fluorescence microscope (EVOS f1, Advanced Microscopy Group, US) at 48 h after transfection. Flow cytometry analyses (CytoFLEX S, Beckman Coulter, US for vectors containing the CMV promoters; CytoFlexSystemB3-R1-V0, Beckman Coulter, US for vectors without the CMV promoters) were performed at 48 h after the transfection to detect the expression of the protein products of ORF1 and ORF2 as indicated by the EGFP and mCherry fluorescence respectively. The data was analyzed with CytExpert software (v2.0.0.153 and v2.4.0.28 for vectors with and without CMV promoters respectively) (Beckman Counter, US). Three independent biological replicates were carried out. The flow cytometry analyses were performed by SyngenTech Corporation (Beijing).

### Ribosome profiling

K562 cells were treated with either 80 μM etoposide or 1 μM imatinib for 0, 6, 24, or 36, then centrifuged at 500*g* for 5 min at 4℃, and resuspended in 1 ml fresh RPMI 1640 medium containing 0.1 mg/ml cycloheximide and incubated at 37℃ in 5% CO_2_ for 1 min. Cells were then centrifuged at 500*g* for 5 min at 4℃, and resuspended in 1 ml pre-cold PBS containing 0.1 mg/ml cycloheximide, and centrifuged at the same condition. After removal of the supernatants, cell pellets were frozen immediately by liquid nitrogen for >1 min. Two biological replicates of 36 h etoposide-treated samples were used for Ribo-seq at GENE DENOVO Corporation (Guangzhou) at 100-million-read scale. Equal amounts of 0 h drug-treated cells and 6, 24, or 36 h etoposide or imatinib-treated cells were pooled and subjected to Ribo-seq at Novogene Corporation (Beijing) at 30-million-read scale.

### Supplementary Information

**Additional file 1: Figures S1-S12. Fig. S1. **Pipeline of sequencing library construction and bioinformatics analysis. **Fig. S2. **Ratio of real vs simulated SICER clusters and CACFs based on 100 simulations in the 4 phenotypic assay systems. **Fig. S3. **Structures of transcripts harboring a recently annotated InSETe in *KHK* locus. **Fig. S4. **Example of novel transcripts representing retained intron of *TPCN2* gene. **Fig. S5. **Structures of all detected transcripts sharing novel intragenic InSETe in *ESYT2* locus. **Fig. S6.** Structures and expression analysis of transcripts containing novel intragenic InSETe in *DNAH8* locus. **Fig. S7. **Structures and expression analyses of transcripts containing different types of novel intragenic InSETes. **Fig. S8. **Structures of transcripts representing novel 3′ extension of known protein-coding gene *LYL6*. **Fig. S9. **Sequence and in silico translation of the InSETT in the *MAML2* locus selected for the in vivo protein coding analysis. **Fig. S10.** Sequence and in silico translation of the InSETT in the *ESYT2* locus selected for the in vivo protein coding analysis. **Fig. S11.** In vivo characterization of the protein coding potential of *MAML2* and *ESYT2* novel transcripts. **Fig. S12.** Expression of the ORF1 and ORF2 products from the novel *MAML2* or *ESYT2* transcript depends on promoters upstream of ORF1.

**Additional file 2: Tables S1-S24. Table S1. **Comparison with CRISPR/Cas9 screen on known genes. **Table S2. **Average TPM. **Table S3. **Comparison with CRISPR/Cas9 screen on lncRNAs. **Table S4.** Number of IACFs or non-phenotypic insertions in different genomic elements. **Table S5.** Clusters generated from simulated positions. **Table S6.** CACFs obtained from simulated positions. **Table S7. **Number of CACFs in different genomic elements. **Table S8. **Odds ratio of IACFs or non-phenotypic insertions in different genomic elements. **Table S9.** Odds ratio of insertions inside or outside of the CACFs in different genomic elements.** Table S10. **Conservation scores of the phenotypic vs non-phenotypic insertions based on IACFs or insertions located inside or outside of the CACFs. **Table S11. **InSETe information. **Table S12.** InSETT sequencing results summary. **Table S13. **InSETG sequencing results summary. **Table S14. **Expression of novel genes (InSETGs) or novel transcripts (InSETTs or InSETes) in normal human tissues and primary cells based on the FANTOM 5 CAGE data. **Table S15.** Distribution of real and simulated TSS clusters in the genome. **Table S16.** Number of TSS clusters in the real and simulated data with or without overlapping CAGE tags in at least one human tissue or primary cell sample. **Table S17. **CAGE signal in real and simulated InSET TSS clusters. **Table S18.** RT-qPCR analysis of InSETTs and InSETGs. **Table S19.** RT-qPCR analysis of all InSETTs and InSETGs. **Table S20.** The log_2_ cytosol/nucleus ratio of the novel and annotated transcripts of *MAML2* and *ESYT2*. **Table S21.** RT-PCR primers. **Table S22.** RACE primers. **Table S23.** RT-qPCR primers of InSETTs. **Table S24.** RT-qPCR primers of InSETGs.

## Data Availability

All data generated or analyzed during this study are included in this published article, its supplementary information files and publicly available repositories. The NGS data were submitted to GEO with accession number GSE220164 [89]. Processed data are presented in Additional file 2: Supplementary Tables and referred to in the appropriate places in the main text, figure legends, and Methods.

## Abbreviations

AI: Artificial intelligence
CACF: Cluster affecting cellular fitness
CAGE: Cap analysis of gene expression
CCS: Circular consensus sequencing
CS: CRISPR score
eRNA: Enhancer RNA
FDR: False discovery rate
GB: Giga-base
gRNA: Guide RNA
GS: Exon-specific primer
GSN: Exon-specific nested primer
IACF: Insertion affecting cellular fitness
InSET: Insertion-based Screen for functional Elements and Transcripts
InSETe: InSET exon
InSETG: InSET gene
InSETT: InSET transcript
LncRNA: Long non-coding RNA
NMD: Nonsense-mediated decay
ORF: Open reading frame
PE150: Paired-end 150 bp
RACE: Rapid amplification of cDNA ends
rcGS: Reverse complement GS primer
rcGSN: Reverse complement GSN primer
sgRNA: Single-guide RNA
SS: Screen score
TPM: Transcripts per million
TSS: Transcription start site
VlincRNA: Very long intergenic non-coding RNA
