# Supplementary material for: Evidence for widespread existence of functional novel and non-canonical human transcripts
Source: BMC Biol. 2023 Nov 24;21:271. doi: 10.1186/s12915-023-01753-5 (PMC10675921; doi:10.1186/s12915-023-01753-5)
Supplement: Supplementary file 1 — Additional file 1: Figures S1-S12. Fig. S1. Pipeline of sequencing library construction and bioinformatics analysis. Fig. S2. Ratio of real vs simulated SICER clusters and CACFs based on 100 simulations in the 4 phenotypic assay systems. Fig. S3. Structures of transcripts harboring a recently annotated InSETe in KHK locus. Fig. S4. Example of novel transcripts representing retained intron of TPCN2 gene. Fig. S5. Structures of all detected transcripts sharing novel intragenic InSETe in ESYT2 locus. Fig. S6. Structures and expression analysis of transcripts containing novel intragenic InSETe in DNAH8 locus. Fig. S7. Structures and expression analyses of transcripts containing different types of novel intragenic InSETes. Fig. S8. Structures of transcripts representing novel 3′ extension of known protein-coding gene LYL6. Fig. S9. Sequence and in silico translation of the InSETT in the MAML2 locus selected for the in vivo protein coding analysis. Fig. S10. Sequence and in silico translation of the InSETT in the ESYT2 locus selected for the in vivo protein coding analysis. Fig. S11. In vivo characterization of the protein coding potential of MAML2 and ESYT2 novel transcripts. Fig. S12. Expression of the ORF1 and ORF2 products from the novel MAML2 or ESYT2 transcript depends on promoters upstream of ORF1. [file 12915_2023_1753_MOESM1_ESM.pdf]

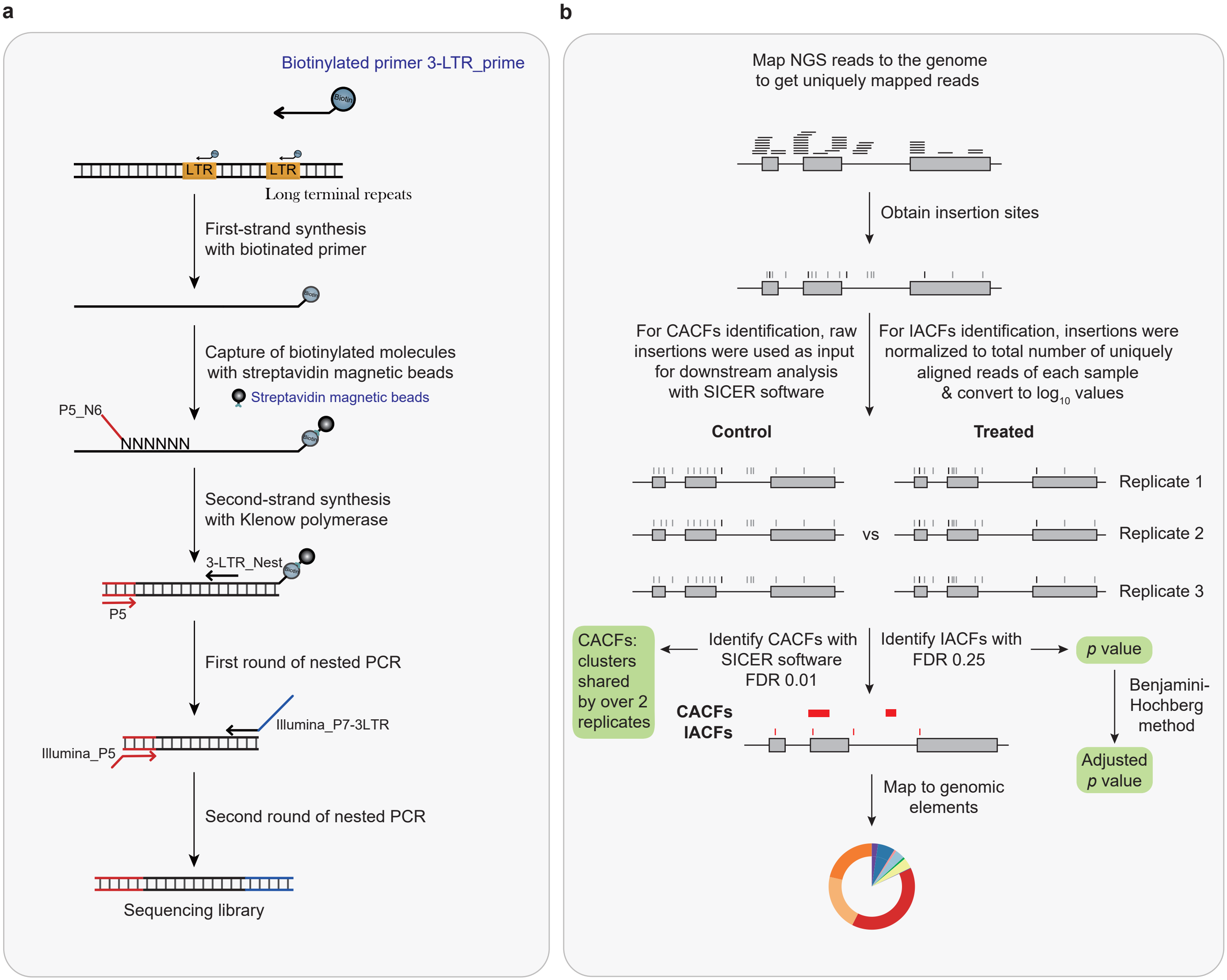

Figure S1. Pipeline of sequencing library construction and bioinformatics analysis. a, Diagram of the sequencing library preparation for detection of lentiviral insertions. **b,** Analytical pipeline for detection and analysis of IACFs and CACFs.

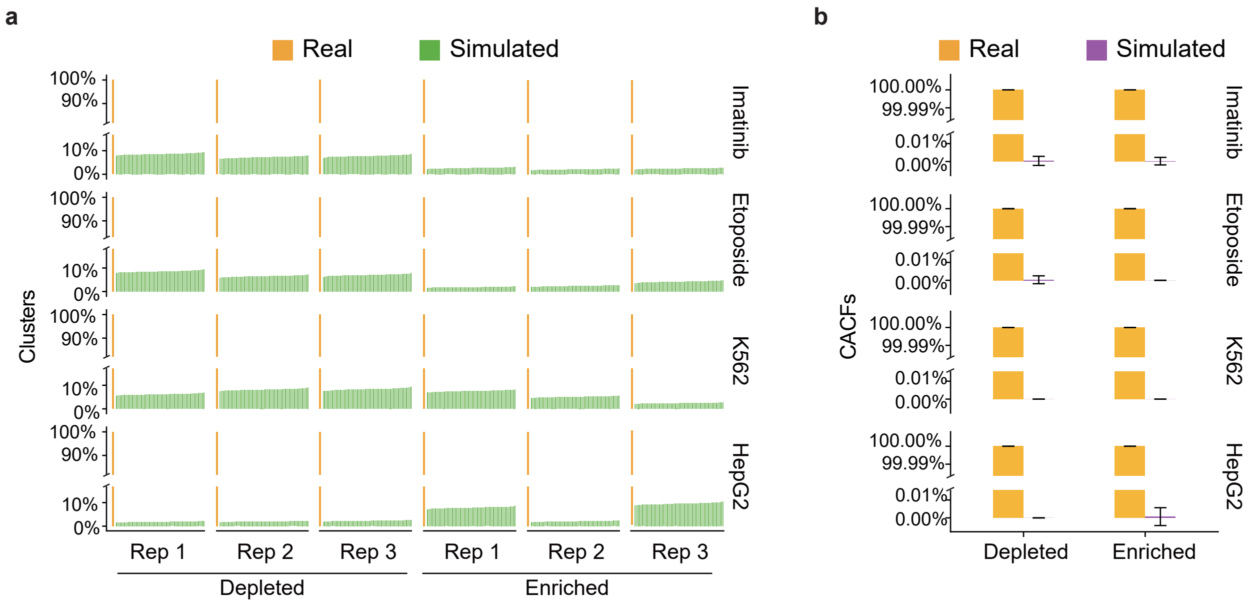

Figure S2. Ratio of real vs simulated SICER clusters (a) and CACFs (b) based on 100 simulations in the 4 phenotypic assay systems. Simulations for 3 individual biological replicates for each system are shown. In panel **b**, error bars indicate SD. Source data are provided in Additional file 2: Tables S5 and S6.

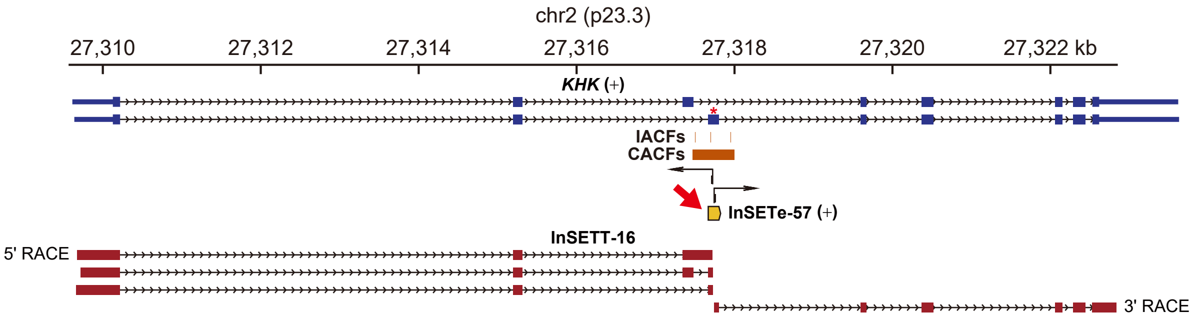

Figure S3. Structures of transcripts harboring a recently annotated InSETe in *KHK* locus. Known gene annotations are in blue and based on the GENCODE Genes track from the UCSC Genome Browser. The recently annotated exon is marked by asterisk. The InSETe is in yellow and marked by the red arrow. Transcript structures obtained from 5'/3' RACE and Nanopore sequencing are shown in red. IACFs and CACFs are in brown and represent unique insertions and merged clusters from only the drug survival system. Black arrows indicate RACE primers.

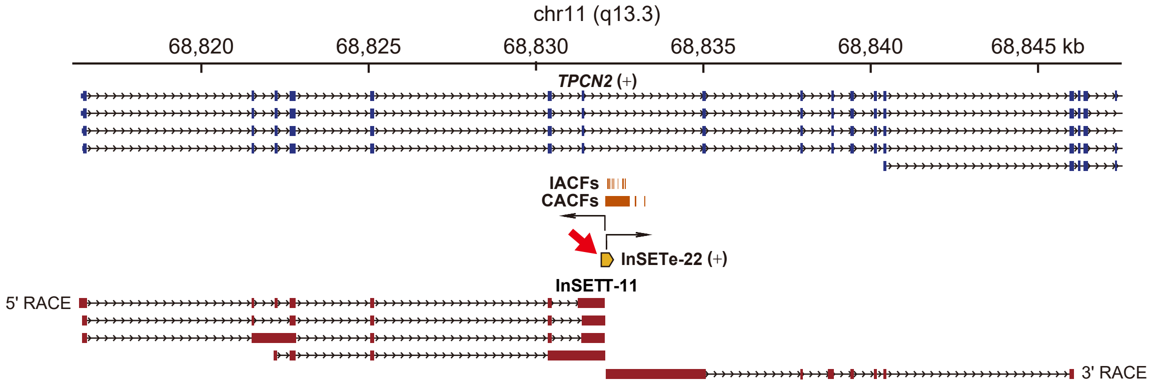

Figure S4. Example of novel transcripts representing retained intron of *TPCN2* gene. Known gene annotations are in blue and based on the GENCODE Genes track from the UCSC Genome Browser. The InSETe is in yellow and marked by the red arrow. Transcript structures obtained from 5'/3' RACE and Nanopore sequencing are shown in red. IACFs and CACFs are in brown and represent unique insertions and merged clusters from only the drug survival system. Black arrows indicate RACE primers.

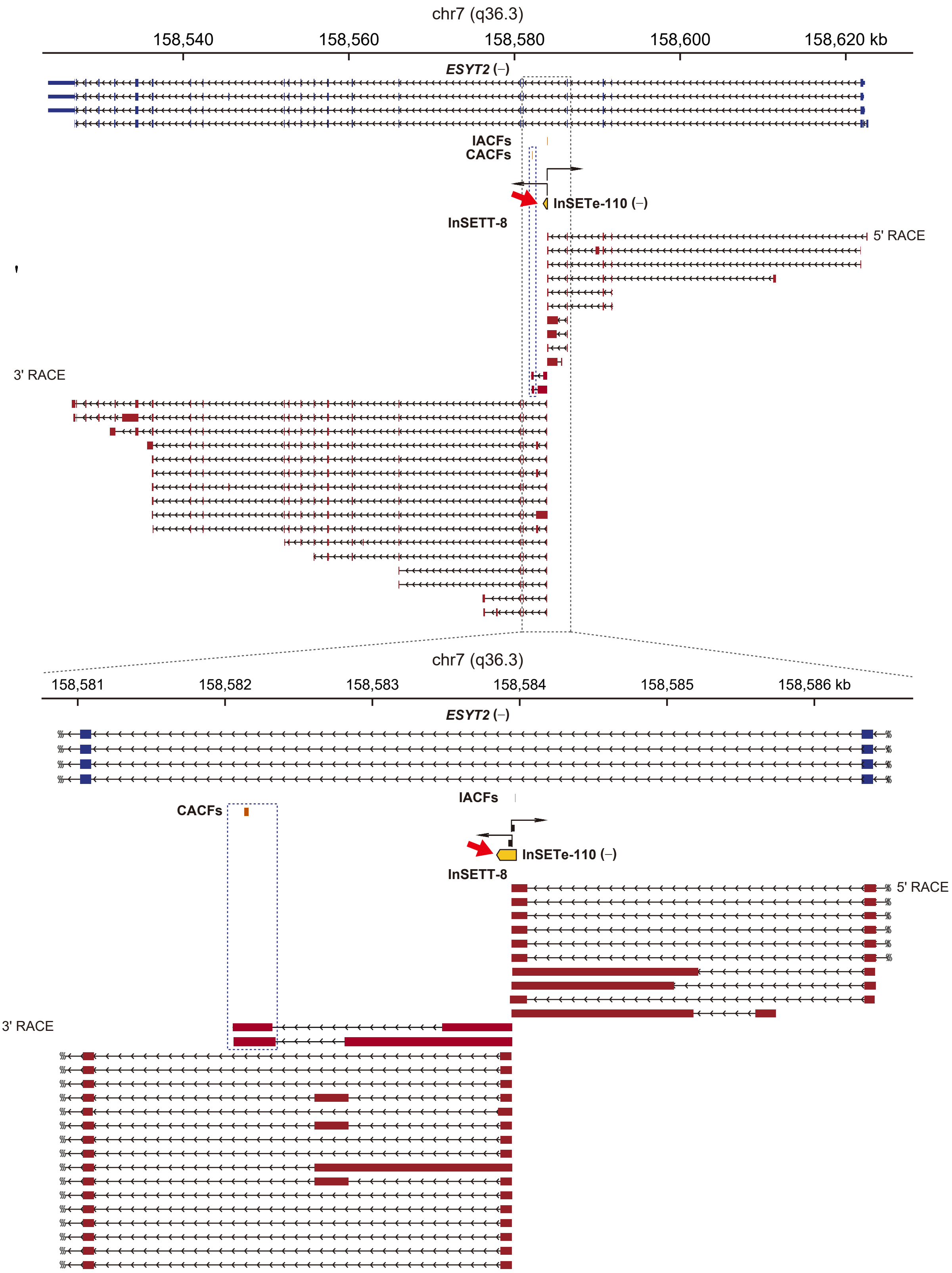

Figure S5. Structures of all detected transcripts sharing novel intragenic InSETe in *ESYT2* locus. The bottom portion represents zoom-in view of the boxed region above. Known genes annotations are in blue and based on the GENCODE Genes track from the UCSC Genome Browser. The InSETe-110 inside *ESYT2* gene is in yellow and marked by the red arrow. Structures of InSETT-8 cluster of novel transcripts (sharing InSETe-110) obtained from 5'/3' RACE and Nanopore sequencing are shown in red. IACFs and CACFs are in brown and represent unique insertions and merged clusters from only the drug survival system. Blue dotted rectangle marks the CACF that overlaps with two novel exons that were neither present in the GENCODE annotations nor detected by the GENSCAN program, but identified by RACE. Black arrows indicate RACE primers.

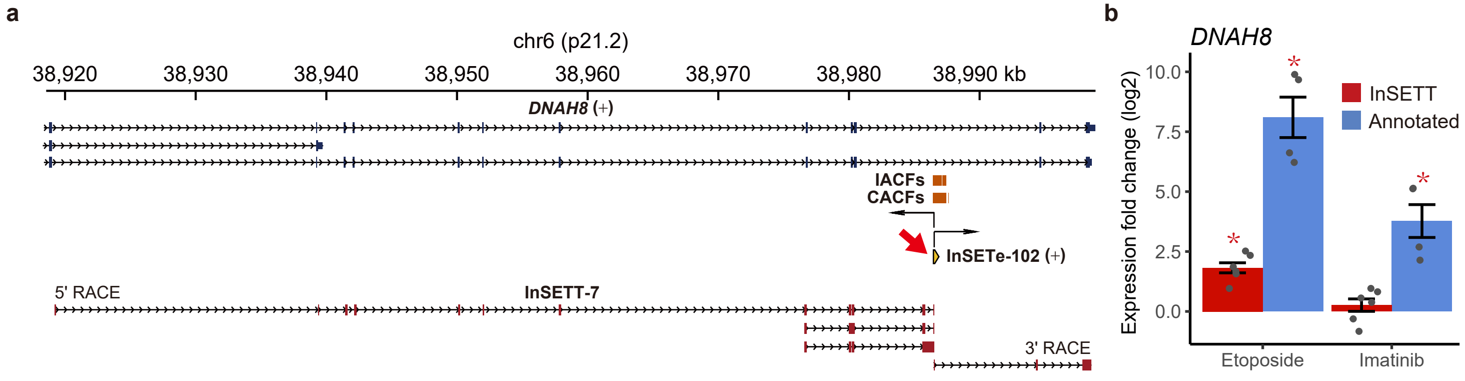

Figure S6. Structures and expression analysis of transcripts containing novel intragenic InSETe in *DNAH8* locus. a, Nanopore sequencing results of 5'/3' RACE products for novel exon inside *DNAH8* gene. Known genes annotations are in blue and based on the GENCODE Genes track from the UCSC Genome Browser. The InSETe is in yellow and marked by the red arrow. Transcript structures obtained from 5'/3' RACE and Nanopore sequencing are shown in red. IACFs and CACFs are in brown and represent unique insertions and merged clusters from only the drug survival system. Black arrows indicate the RACE primers. **b,** The $\log_2$ expression fold changes (Y-axis) of *DNAH8* novel and annotated transcripts under etoposide or imatinib treatment compared to the DMSO treated control are shown. Error bars indicate the SE of a total of 6 technical replicates in 2 biological replicates. Asterisks indicate significant differences under two-sided paired Student's *t*-test (*p value* < 0.05). Source data are provided in Additional file 2: Table S18.

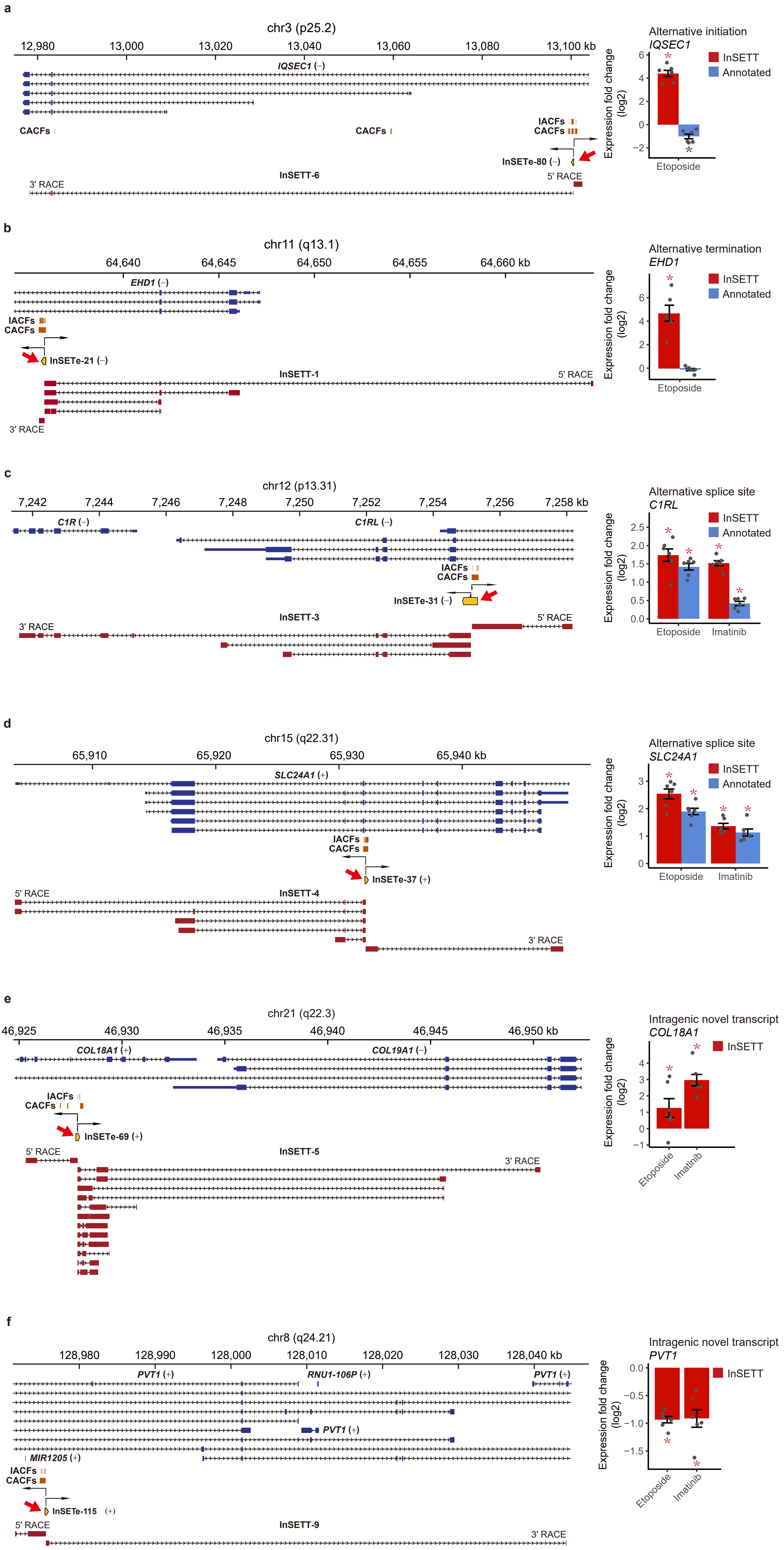

Figure S7. Structures and expression analyses of transcripts containing different types of novel intragenic InSETes. For each panel, genomic view is shown on the left and expression analysis is shown on the right. **a, b,** InSETes representing alternative initiation (**a**) or termination (**b**) are shown. **c, d,** InSETes representing alternative splice sites are shown. **e, f,** InSETes representing intragenic novel transcripts without evidence of connection to the known genes are shown. Known genes annotations are in blue and based on the GENCODE Genes track from the UCSC Genome Browser. The InSETes are in yellow and marked by the red arrows. Transcript structures obtained from 5'/3' RACE and Nanopore sequencing are shown in red. IACFs and CACFs are in brown and represent unique insertions and merged clusters from only the drug survival system. Black arrows indicate the RACE primers. The expression analysis shows the $\log_2$ fold changes (Y-axes) of the novel, and annotated transcripts if the novel exons were connected to annotated genes, for each respective locus under etoposide or imatinib treatment compared to the DMSO treated control. Error bars indicate the SE of a total of 6 technical replicates in 2 biological replicates. Asterisks indicate significant differences under two-sided paired Student's *t*-test (*p value* < 0.05). Source data are provided in Additional file 2: Table S18.

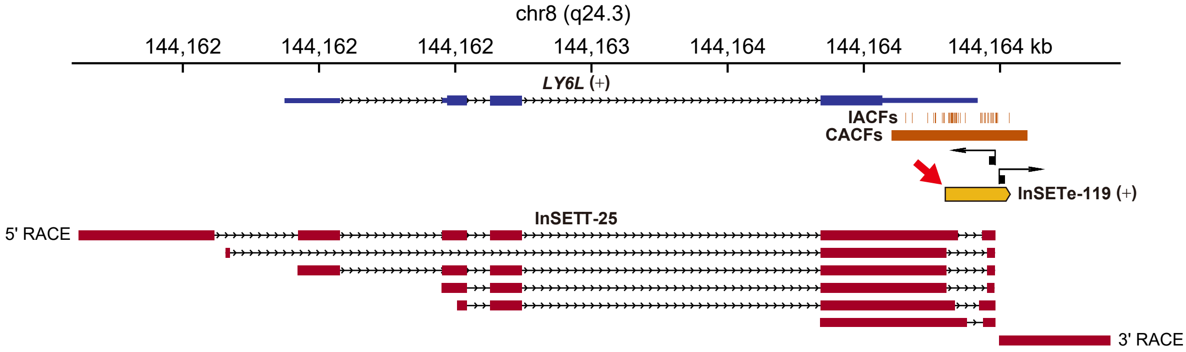

Figure S8. Structures of transcripts representing novel 3' extension of known protein-coding gene *LYL6*. Known gene annotation is in blue and based on the GENCODE Genes track from the UCSC Genome Browser. The InSETe is in yellow and marked by the red arrow. Transcript structures obtained from 5'/3' RACE and Nanopore sequencing are shown in red. IACFs and CACFs are in brown and represent unique insertions and merged clusters from only the drug survival system. Black arrows indicate RACE primers.

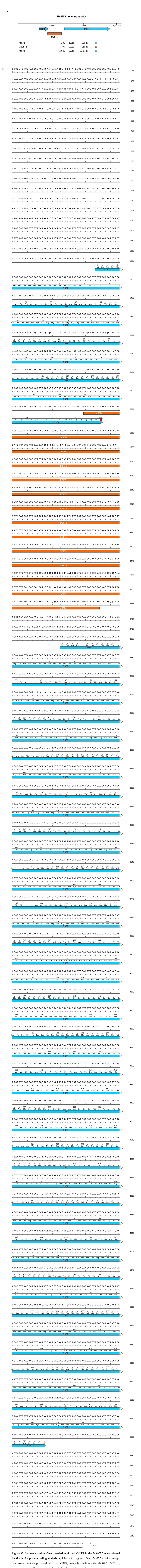

Figure S9. Sequence and *in silico* translation of the InSETT in the *MAML2* locus selected for the *in vivo* protein coding analysis. a, Schematic diagram of the *MAML2* novel transcript. Blue arrows indicate predicted ORF1 and ORF2, orange box indicates the *MAML2* InSETe. **b,** Sequence and *in silico* translation of the *MAML2* novel transcript.

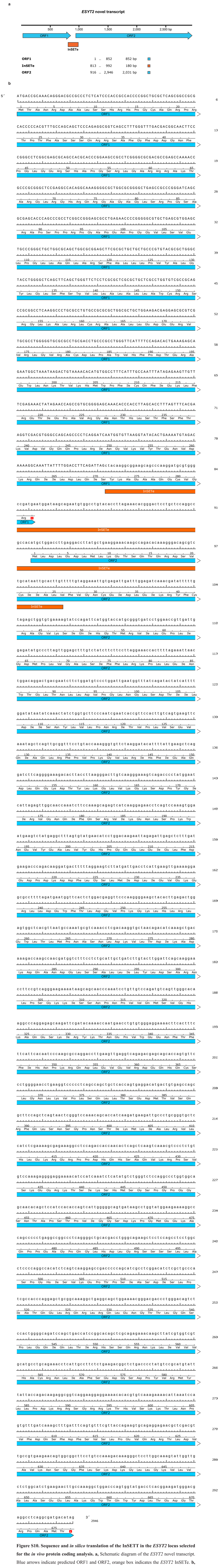

Figure S10. Sequence and *in silico* translation of the InSETT in the *ESYT2* locus selected for the *in vivo* protein coding analysis. a, Schematic diagram of the *ESYT2* novel transcript. Blue arrows indicate predicted ORF1 and ORF2, orange box indicates the *ESYT2* InSETe. **b,** Sequence and *in silico* translation of the *ESYT2* novel transcript.

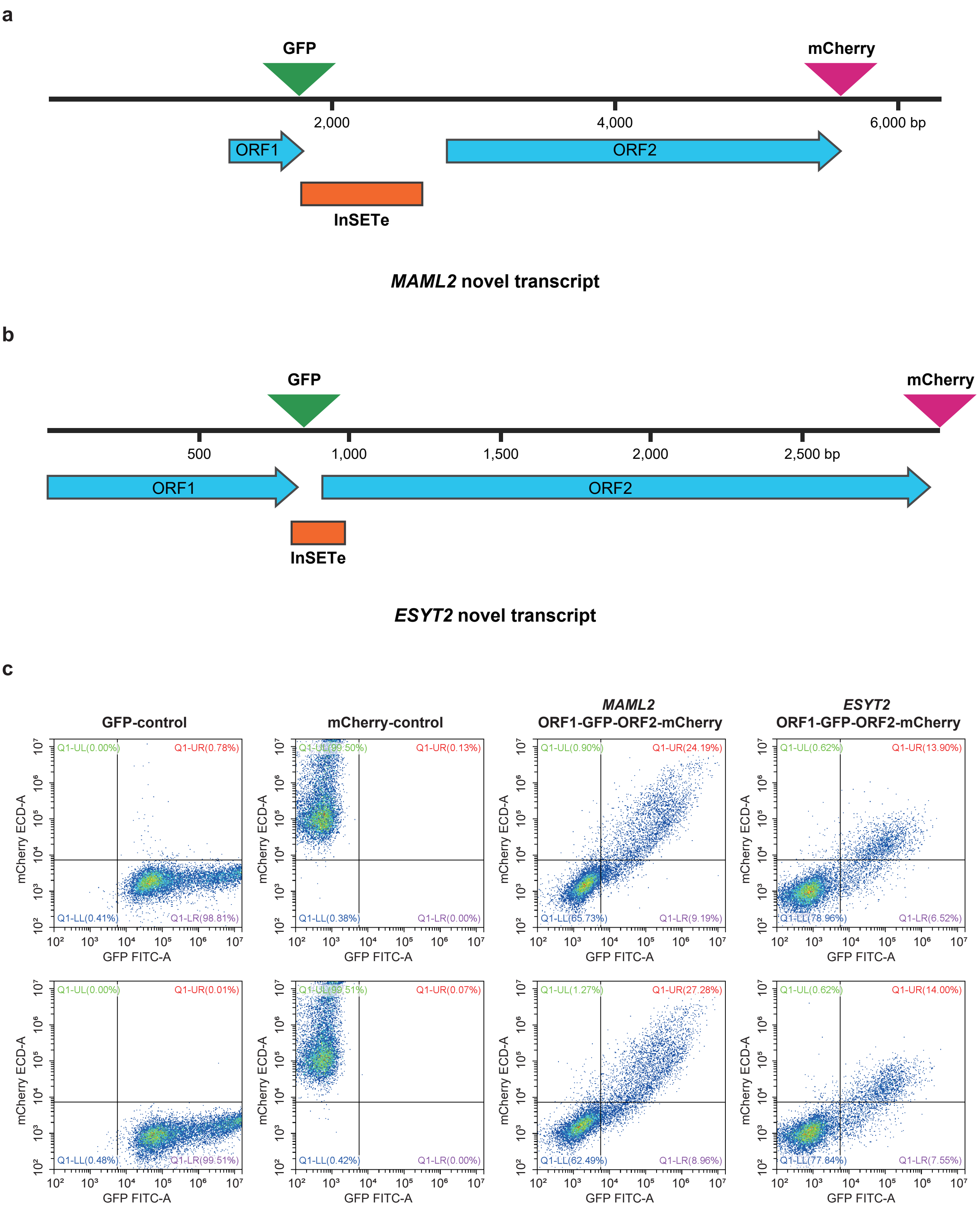

Figure S11. *In vivo* characterization of the protein coding potential of *MAML2* and *ESYT2* novel transcripts. a, b, Schematic diagram of *in-silico* predicted major ORFs in the InSETTs formed by the inclusion of the novel cassette InSETes in the *MAML2* and *ESYT2* loci chosen for these experiments, and positions of in-frame fusions of the GFP and mCherry proteins in those ORFs. Blue arrows indicate predicted ORF1 and ORF2, orange boxes indicate the novel exons. Green and magenta triangles indicate the position where GFP and mCherry proteins were inserted. **c,** Flow cytometry analysis results of 293FT cells transfected with vectors harboring the GFP/mCherry fusions for the *MAML2* or *ESYT2* novel transcript shown in panels **a** and **b** with GFP or mCherry inserted in-frame after the predicted ORF1 and ORF2 respectively. 293FT cells without transfection and those transfected with vectors expressing either GFP or mCherry only serve as the negative and positive controls respectively. Two biological replicates in addition to the one in Fig. 8e are shown.

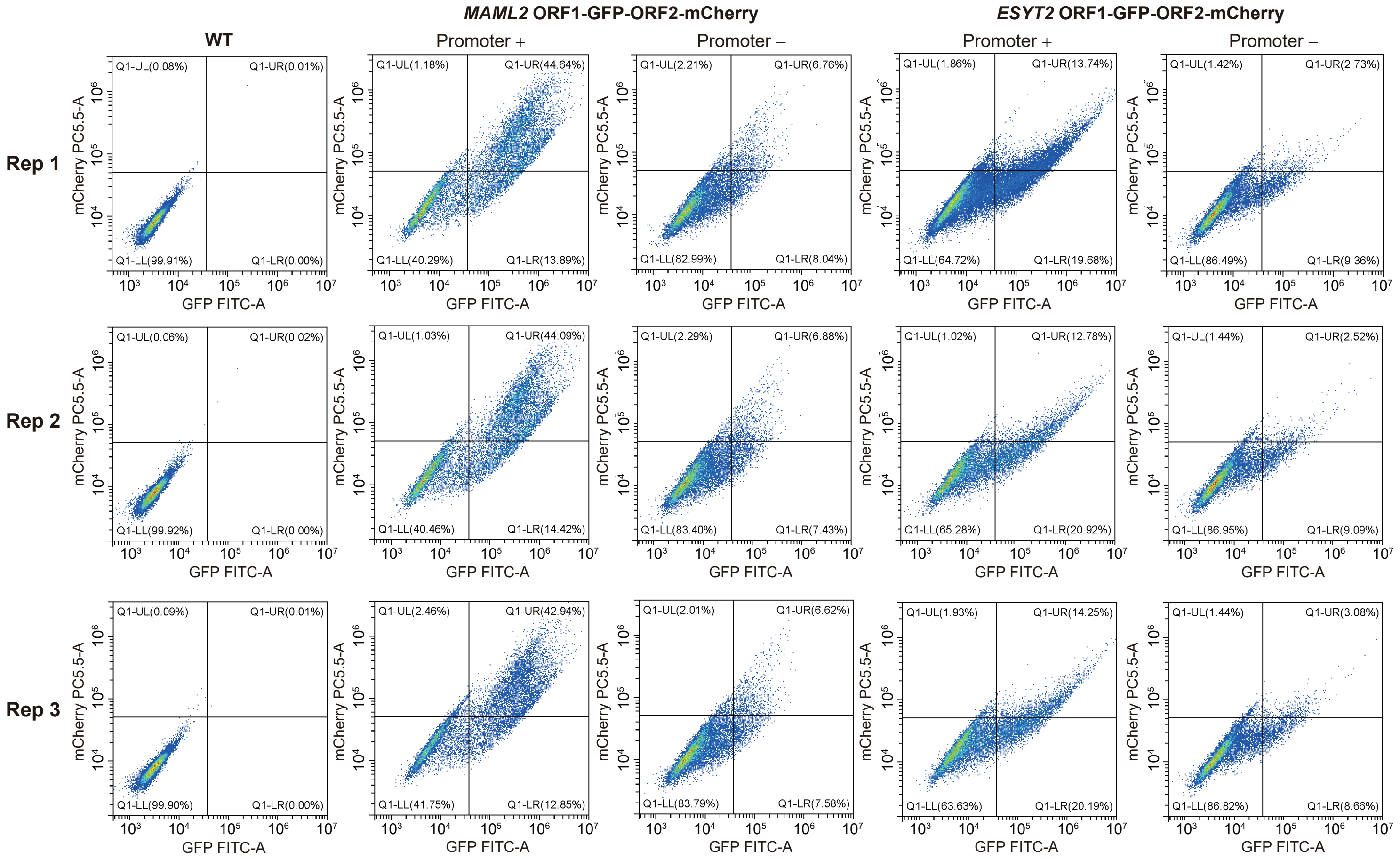

Figure S12. Expression of the ORF1 and ORF2 products from the novel *MAML2* or *ESYT2* transcript depends on promoters upstream of ORF1. The figure shows results of the flow cytometry analyses of 293FT cells transfected with plasmids harboring the GFP/mCherry fusions and the corresponding controls without the CMV promoter sequences upstream of ORF1 for each gene and from each of the 3 biological replicates.
